# Genes involved in TGFβ1-driven epithelial-mesenchymal transition of renal epithelial cells are topologically related in the human interactome map

**DOI:** 10.1186/1471-2164-8-383

**Published:** 2007-10-22

**Authors:** Stefano Campanaro, Simone Picelli, Rossella Torregrossa, Laura Colluto, Monica Ceol, Dorella Del Prete, Angela D'Angelo, Giorgio Valle, Franca Anglani

**Affiliations:** 1 CRIBI Biotechnology Center, Department of Biology, University of Padova, Italy; 2 Laboratory of Histomorphology and Molecular Biology of the Kidney, Department of Medical and Surgical Sciences, Division of Nephrology, University of Padova, Italy

## Abstract

**Background:**

Understanding how mesenchymal cells arise from epithelial cells could have a strong impact in unveiling mechanisms of epithelial cell plasticity underlying kidney regeneration and repair.

In primary human tubular epithelial cells (HUTEC) under different TGFβ1 concentrations we had observed epithelial-to-mesenchymal transition (EMT) but not epithelial-myofibroblast transdifferentiation. We hypothesized that the process triggered by TGFβ1 could be a dedifferentiation event. The purpose of this study is to comprehensively delineate genetic programs associated with TGFβ1-driven EMT in our in vitro model using gene expression profile on large-scale oligonucleotide microarrays.

**Results:**

In HUTEC under TGFβ1 stimulus, 977 genes were found differentially expressed. Thirty genes were identified whose expression depended directly on TGFβ1 concentration. By mapping the differentially expressed genes in the Human Interactome Map using Cytoscape software, we identified a single scale-free network consisting of 2630 interacting proteins and containing 449 differentially expressed proteins. We identified 27 hub proteins in the interactome with more than 29 edges incident on them and encoded by differentially expressed genes. The Gene Ontology analysis showed an excess of up-regulated proteins involved in biological processes, such as "morphogenesis", "cell fate determination" and "regulation of development", and the most up-regulated genes belonged to these categories. In addition, 267 genes were mapped to the KEGG pathways and 14 pathways with more than nine differentially expressed genes were identified. In our model, Smad signaling was not the TGFβ1 action effector; instead, the engagement of RAS/MAPK signaling pathway seems mainly to regulate genes involved in the cell cycle and proliferation/apoptosis.

**Conclusion:**

Our present findings support the hypothesis that context-dependent EMT generated in our model by TGFβ1 might be the outcome of a dedifferentiation. In fact: 1) the principal biological categories involved in the process concern morphogenesis and development; 2) the most up-regulated genes belong to these categories; and, finally, 3) some intracellular pathways are involved, whose engagement during kidney development and nephrogenesis is well known. These long-term effects of TGFβ1 in HUTEC involve genes that are highly interconnected, thereby generating a scale-free network that we named the "TGFβ1 interactome", whose hubs represent proteins that may have a crucial role for HUTEC in response to TGFβ1.

## Background

Epithelial-to-mesenchymal transition (EMT) of renal tubular cells is a fundamental sign of epithelial cell plasticity in physiological processes such as regeneration and wound healing, but it also characterizes pathological conditions such as fibrosis and carcinogenesis.

The adult mammalian renal tubular epithelium exists in a relatively quiescent to slowly replicating state, but has great potential for regenerative morphogenesis following severe ischemic or toxic injury [1]. Dedifferentiation, i.e. the acquisition of mesenchymal markers such as vimentin and N-cadherin, seems to represent a crucial step in the recovery of tubular integrity and precedes the reconstitution of a well-differentiated morphology. In the adult kidney, however, the tubular cells' acquisition of a mesenchymal phenotype represents one of the crucial steps towards transdifferentiation into myofibroblasts, the effector cells of tubulo-interstitial fibrosis [2].

Transforming growth factor β1 (TGFβ1) is a key modulator of EMT in a variety of epithelial cells, but is also capable of inducing the myofibroblast phenotype, i.e. the acquisition of alpha smooth muscle actin (αSMA) microfilaments in fibroblasts during wound healing, in mesangial cells in culture and in renal tubular cells [3].

TGFβ1-induced EMT appears to depend primarily on intact Smad signaling. To date, Smad proteins are the only TGFβ1 receptor substrates with a demonstrated ability to propagate signals [4]. It is now becoming evident, however, that EMT is not a uniform process. Its role and features clearly differ, depending on the physiological context and type of epithelia (developmental EMT, oncogenic EMT, non-oncogenic EMT) [5].

Using primary human tubular epithelial cells (HUTEC), we demonstrated that chronic exposure to TGFβ1 prompted morphological, molecular and biochemical changes towards a mesenchymal phenotype, but this gave rise to no de novo expression of αSMA gene or myofibroblast phenotype [6]. We hypothesized that the process triggered by TGFβ1 in our model is a dedifferentiation event that may be part of the vital plasticity of renal tubular cells.

Our results prompted us to further characterize this EMT process. Since microarray technology powerfully monitors gene expression and has led to the discovery of pathways regulating complex biological processes, we explored the molecular mechanisms underlying this transition using this approach. A global view of the EMT process was obtained identifying the Gene Ontology (GO) classes enriched by differentially expressed genes and analyzing KEGG pathways involved in signal transduction. To obtain an overview of their topological properties, we also mapped differentially expressed proteins in the human interactome map using Cytoscape software. This analysis enabled us to establish that about 50% of the genes up- and down-regulated by TGFβ1 were strongly interconnected and formed a large network that we named the "TGFβ1 interactome".

## Results

At genome-wide level, we investigated the expression profile changes occurring in the EMT of primary HUTEC under chronic TGFβ1 treatment. Our in vitro model of human renal EMT has been described in detail elsewhere [6]. By immunocytochemistry, we demonstrated that, in addition to the front-end to back-end cell morphology, TGFβ1 can induce a markedly dose-dependent up-regulated expression of mesenchymal markers, including collagen III, with a parallel drop in the expression of epithelial markers such as E-cadherin and cytokeratin. In our in vitro model, however, TGFβ1 stimulation did not induce conversion to the myofibroblast phenotype [6].

Cultures treated for 4 days with 5–10–50 ng/ml of TGFβ1 were considered for the microarray studies. We refer to these experiments as "coll-5 ng", "coll-10 ng", "coll-50 ng", "plast-5 ng", "plast-10 ng" and "plast 50 ng". To ascertain the role of the substrate, the expression profile of cells cultured on plastic plates was also compared with that of cells grown on collagen I in basal conditions; we refer to this experiment as "coll-vs-plast".

Each of the seven experiments was performed in duplicate, differentially expressed genes were obtained independently for each comparison and results are given in the Additional file 1. Of ~21000 unique genes of the human oligo chip, we found 977 differentially expressed, represented by 993 spots (Additional file 1).

**Figure 1:**
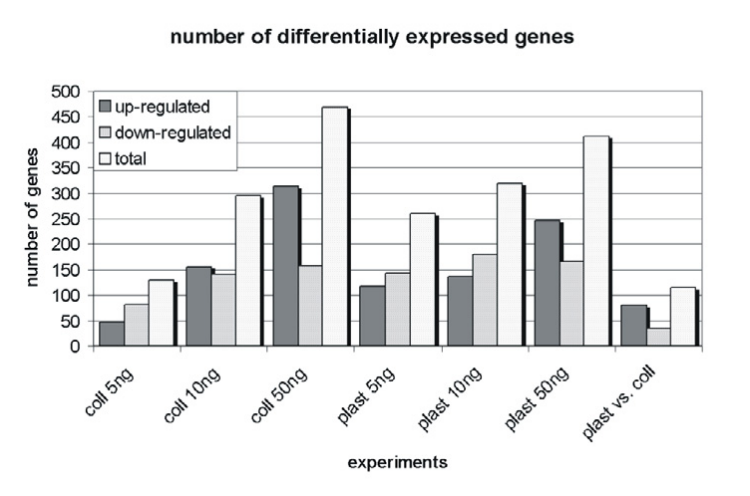
**Number of differentially expressed genes in each experimental condition**. Cells were grown on plastic (plast) or collagen I (coll) plates and exposed to different TGFβ1 concentrations (5–10–50 ng/ml). The last experiment (plast vs. coll) is the comparison between cells grown on the two different substrates and not treated with TGFβ1.

Since our experiment had a typical dose-response design, the expected effect was a dose-related increase in differentially expressed genes, as shown in Figure 1. Only the up-regulated genes increased considerably, however, going from 5 to 50 ng/ml of TGFβ1, while the down-regulated genes remained much the same in this range. This result did not depend on the cell-growing substrate, though it was more prominent for collagen I. Probably the higher the TGFβ1 concentration, the greater the number of genes exceeding the significance threshold.

**Figure 2:**
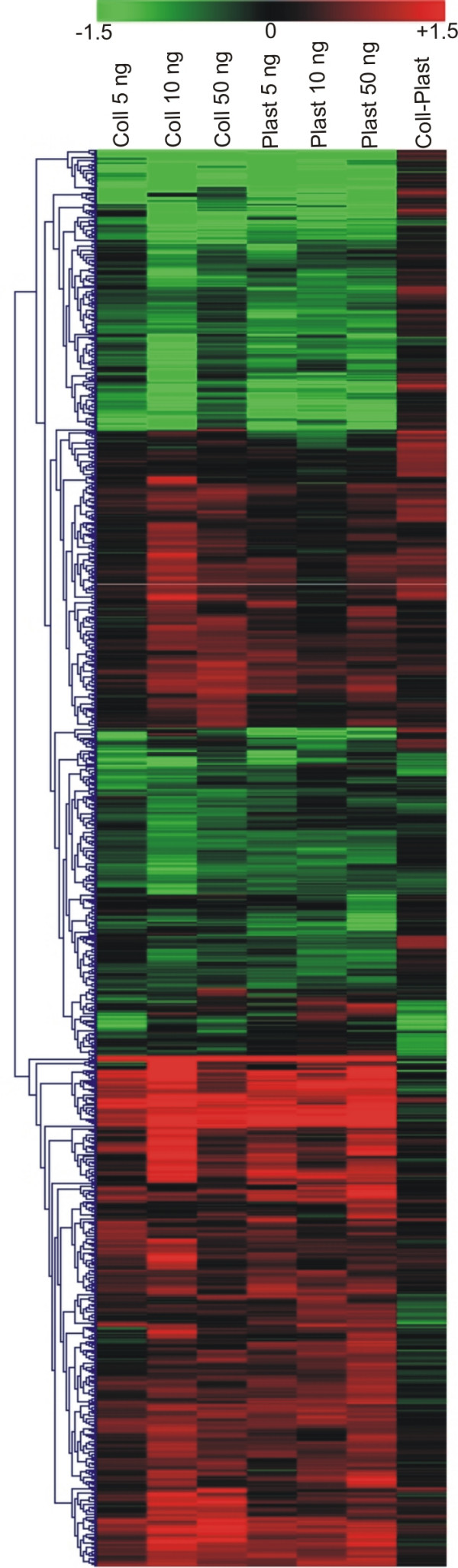
**Cluster analysis of the differentially expressed genes using TMEV software**. The 993 genes in Additional file 1 are clustered on the basis of the log_2 _expression ratio. Up-regulated and down-regulated genes are colored in red and green, respectively. Columns refer to the seven experiments performed using different TGFβ1 concentrations (5–10–50 ng/ml) and substrates (collagen type I and plastic). Note that genes are grouped in large blocks, in which they tend to be up- or down-regulated in all the first six experiments.

To establish whether the differentially expressed genes in each experiment were coordinately regulated, we compared expression ratios using cluster analysis (Figure 2). We identified four clusters of coordinately regulated genes that were common to the six experiments, demonstrating that the expression profile is similar for all the TGFβ1 concentrations whatever the substrate used. This finding also means that no illegitimate induction took place and seems to support the suggestion that more differentially expressed genes are obtained with the higher dosages because more genes exceed the threshold, not because there are more "new" genes.

Given these results, a global list of 919 differentially expressed genes, excluding those identified in the "coll-vs-plast" experiment, was considered for the GO, KEGG and other analyses.

We performed a statistical analysis using the SAM software to assign a significance level to the genes whose expression depended on the TGFβ1 dosage. We identified 25 genes related directly and five genes related inversely to the TGFβ1 dosage with a q value of zero, indicating a strong correlation between dosages and expression levels. The complete list of the genes is given in Table 1. To avoid false negatives, we applied very stringent criteria in the statistical analysis, selecting only genes with a q value of zero – hence their small number. Five of these genes were also identified by Zavadil et al. [7] in time-dependent microarray experiments on human keratinocytes. THBS1, WEE1, PLS3 and ITGAV expression was increased in both our own dose-dependent experiments and in Zavadil's time-course experiments, while MAL expression was decreased, suggesting the existence of a common regulatory mechanism regardless of the cell type.

**Table 1:** Dose response genes identified using SAM software.

**GB accession ID**	**EntrezGene ID**	**Gene Symbol**	**Description**	**Score(d)**	**R^2**
NM_003246	7057	THBS1	thrombospondin 1	**2,75**	**0.89**
NM_005630	6578	SLCO2A1	solute carrier organic anion transporter family, member 2A1	**2,64**	**0.77**
X62048	7465	WEE1	WEE1 homolog (S. pombe)	**2,56**	**0.64**
AY033606	57120	GOPC	golgi associated PDZ and coiled-coil motif containing	**2,54**	**0.72**
NM_014187	29100	HSPC171	HSPC171 protein	**2,47**	**0.96**
NM_002709	5500	PPP1CB	protein phosphatase 1, catalytic subunit, beta isoform	**2,44**	**0.84**
NM_005032	5358	PLS3	plastin 3 (T isoform)	**2,36**	**0.58**
NM_021977	6581	SLC22A3	solute carrier family 22 (extraneuronal monoamine transporter), member 3	**2,36**	**0.99**
AF220030	117854	TRIM6	tripartite motif-containing 6	**2,33**	**0.76**
NM_018695	55914	ERBB2IP	Erbb2 interacting protein	**2,31**	**0.72**
NM_004735	9208	LRRFIP1	leucine rich repeat (in FLII) interacting protein 1	**2,27**	**0.83**
NM_018561	25862	USP49	ubiquitin specific peptidase 49	**2,25**	**0.74**
NM_032812	84898	PLXDC2	plexin domain containing 2	**2,20**	**0.92**
NM_004718	9167	COX7A2L	cytochrome c oxidase subunit VIIa polypeptide 2 like	**2,20**	**0.84**
NM_016651	51339	DACT1	dapper, antagonist of beta-catenin, homolog 1 (Xenopus laevis)	**2,20**	**0.97**
AK056277	152048	FLJ31715	hypothetical protein FLJ31715	**2,20**	**0.67**
NM_004643	8106	PABPN1	poly(A) binding protein, nuclear 1	**2,18**	**0.64**
NM_000876	3482	IGF2R	insulin-like growth factor 2 receptor	**2,17**	**0.97**
AF326917	26053	AUTS2	autism susceptibility candidate 2	**2,16**	**0.64**
NM_003685	8570	KHSRP	KH-type splicing regulatory protein (FUSE binding protein 2)	**2,14**	**0.52**
NM_001300	1316	KLF6	Kruppel-like factor 6	**2,12**	**0.81**
NM_022823	64838	FNDC4	fibronectin type III domain containing 4	**2,12**	**0.70**
NM_004663	8766	RAB11A	RAB11A, member RAS oncogene family	**2,10**	**0.97**
NM_002210	3685	ITGAV	integrin, alpha V (vitronectin receptor, alpha polypeptide, antigen CD51)	**2,08**	**0.62**
BC018067	9364	RAB28	RAB28, member RAS oncogene family	**2,05**	**0.92**
NM_021738	6840	SVIL	supervillin	**-2,65**	**0.68**
AB028981	23348	DOCK9	dedicator of cytokinesis 9	**-2,40**	**0.67**
NM_002993	6372	CXCL6	chemokine (C-X-C motif) ligand 6 (granulocyte chemotactic protein 2)	**-2,37**	**0.21**
NM_002371	4118	MAL	mal, T-cell differentiation protein	**-2,30**	**0.56**
AF402776	114614	BIC	BIC transcript	**-2,30**	**0.41**

The expression of these transcripts depends on the TGFβ1 concentration used. The first 25 genes are directly related to the TGFβ1 dosage (d value higher than zero), the last five are inversely related (d value lower than zero). Columns show: the GenBank accession number, the EntrezGene ID, the Gene symbol, the gene's description, the "d score" calculated by the SAM software, and the correlation coefficient R2.

The large number of genes regulated by TGFβ1 makes it difficult to understand the role of this growth factor in the EMT process using a gene-by-gene study. We opted for an analysis designed to comprehensively delineate the genetic programs associated with EMT in response to TGFβ1.

The biological significance of differentially expressed genes was explored using two databases as resources, i.e. the GO and the Kyoto Encyclopedia of Genes and Genomes (KEGG) maps (see Material and Methods).

**Figure 3:**
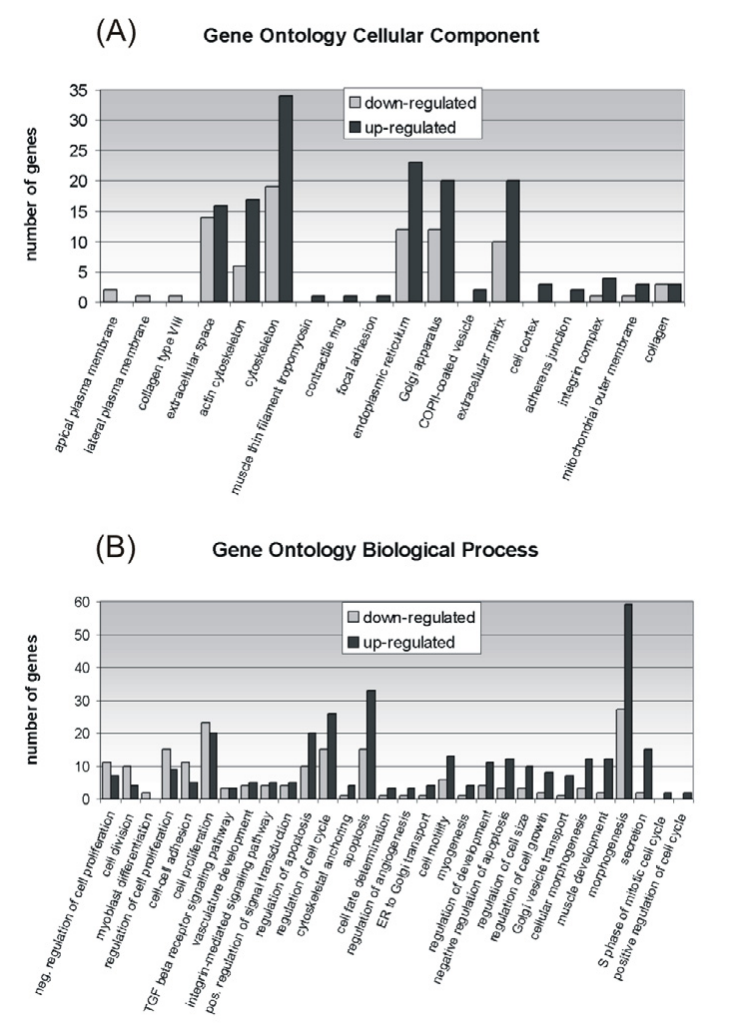
**Gene Ontology annotation of differentially expressed genes**. Up-regulated (black bars) and down-regulated (gray bars) genes shown in Additional file 1 were analyzed using GoMiner software. In (A) and (B), genes are classified respectively on the basis of Biological Process and Cellular Component. Only classes with a p value lower than 0.01 (A) and 0.05 (B) are reported.

Among the categories of biological processes (BP), six featured a large number of down-regulated genes and 20 contained a large number of up-regulated genes (Figure 3B). Among the latter, we found proteins involved in cell proliferation, the cell cycle and cell growth regulation, the cytoskeleton and protein transport. Among the cell component (CC) categories (Figure 3A), there was an over-representation of the up-regulated proteins involved in extracellular matrix modification and cell-cell adhesion and of proteins localized in subcellular compartments of the exocytotic pathway, confirming the importance of these pathways in the EMT reprogramming process. The differentially under-expressed genes belong to the CC class of the apical and lateral plasma membranes, indicating the loss of the typical epithelial morphology and functions of cells undergoing EMT.

Up-regulated genes are particularly over-represented in the BP categories such as "morphogenesis" and "cellular morphogenesis", as well as those belonging to developmental processes such as "cell fate determination", "myogenesis", "regulation of development" and "muscle development", indicating that an embryological program could be awakened.

All the genes containing an EntrezGene ID (886) were mapped to the KEGG database to define the signaling pathways and cellular structures that can participate in the EMT process. Only 267 were classified by the software, nevertheless we identified 14 pathways with more than nine differentially expressed genes that were downloaded and saved (Additional file 2). Up- and down-regulated genes were colored red, orange, blue and light blue (see Material and Methods). A visual inspection of these pathways is useful in clarifying the specific (activator or inhibitor) role of the differentially expressed genes, obtaining more detailed information than from the GO analysis alone. In the "apoptotic" pathway, for instance (Additional file 2), both activators (which are up-regulated) and repressors (which are down-regulated) were identified, providing a clear picture of the cell death process.

The signal transduction pathways involved in EMT were better detailed by KEGG analysis, while other processes such as cell adhesion or cytoskeleton remodeling were also identified using the GO. Seven important intracellular pathways were involved in the EMT process in our model, i.e. TGFβ1/SMAD, MAPK, WNT, JAK/STAT, calcium signaling pathways, the cell cycle and apoptosis.

TGFβ1 is known to activate a large group of genes through both Smad-dependent and Smad-independent pathways [8,9].

We hypothesize that the genes we identified may be closely interconnected in the human interactome, generating a sort of "TGFβ1 interactome". We identified 449 differentially expressed proteins that interact either directly or with undifferentially expressed proteins. The connected component that we highlighted contains 2630 proteins (nodes) and 4183 edges with an averaged connectivity degree of 3.15. The large number of proteins belonging to the cluster identified makes the result noteworthy and indicates that most of the genes regulated by TGFβ1 at transcriptional level during EMT encode proteins that work in a highly coordinated manner.

**Figure 4:**
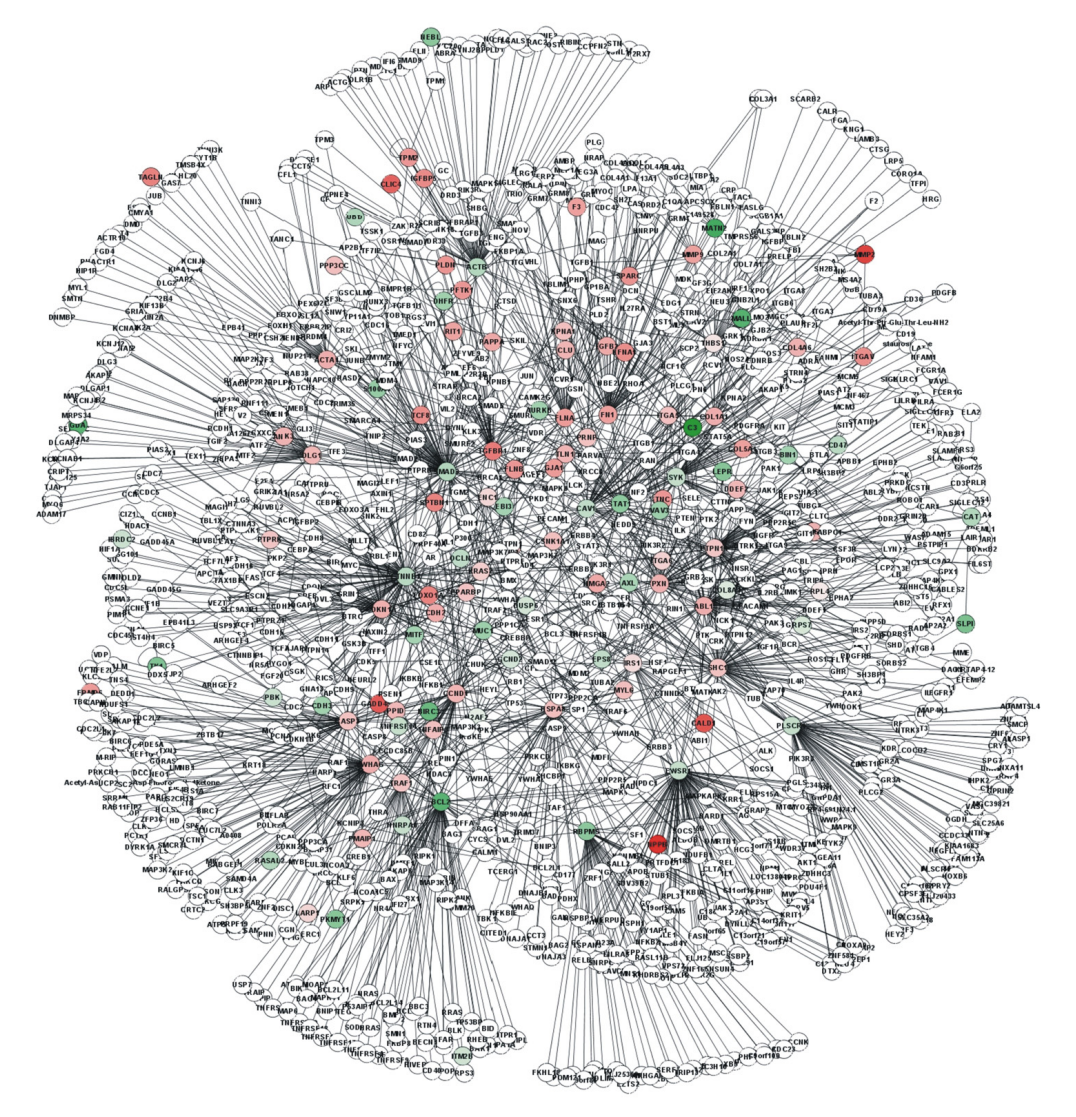
**Cluster of interacting proteins**. The TGFβ1 network consists of 27 hub proteins with more than 29 edges incident on them and proteins that interact directly with them identified in the "TGFβ1 interactome". The hubs are clearly visible in the network from their high degree of connectivity. Proteins encoded by up-regulated and down-regulated genes are 115 and colored respectively in red and green using a color gradient reflecting the mean log_2 _ratio given in Additional file 1, column N. This TGFβ1 core consists of 1235 proteins.

An analysis performed with the tYNA software [10] confirms that the distribution of the connectivity degree in the network follows the power law characteristic of scale-free networks (data not shown). These networks are typical of cell systems and the majority of nodes have few links in such an arrangement, but a few nodes have numerous links, thereby ensuring that the system is fully connected. A few highly-interconnected nodes thus act as hubs that shape the network's overall operation, but may also represent sites of system vulnerability [11]. We selected the proteins with a connectivity degree – i.e. the number of edges incident on them – higher than 29, calling these proteins "hubs". We identified 27 hubs. The complete list of the hubs is given in Table 2. All these proteins were differentially expressed and three of them, PXN, SMAD3 and TGFBR1, interacted with more than 12 differentially expressed proteins. If we consider these 27 hubs and the proteins interacting directly with them, we identify a sort of central core of the TGFβ1 interactome (network) consisting of 1235 proteins (Figure 4), 115 of which were differentially expressed. Using tYNA software, moreover, we found that the hub proteins had the "shortest path length" and were consequently closely connected within the network – in fact, 24 of them interacted directly.

**Table 2:** List of the TGFβ1 interactome hubs.

**GB accession ID**	**EntrezGene ID**	**Gene Symbol**	**Gene description**	**Log ratio**
AK002171	7046	TGFBR1	transforming growth factor, beta receptor I (activin A receptor type II-like kinase, 53 kDa)	**1,04**
NM_001456	2316	FLNA	filamin A, alpha (actin binding protein 280)	**0,77**
NM_002026	2335	FN1	fibronectin 1	**0,73**
NM_001100	58	ACTA1	actin, alpha 1, skeletal muscle	**0,62**
NM_005157	25	ABL1	v-abl Abelson murine leukemia viral oncogene homolog 1	**0,61**
AK024230	7532	YWHAG	tyrosine 3-monooxygenase/tryptophan 5-monooxygenase activation protein, gamma polypeptide	**0,61**
NM_000389	1026	CDKN1A	cyclin-dependent kinase inhibitor 1A (p21, Cip1)	**0,60**
NM_002859	5829	PXN	paxillin	**0,58**
NM_053056	595	CCND1	cyclin D1	**0,55**
NM_004087	1739	DLG1	discs, large homolog 1 (Drosophila)	**0,54**
NM_006597	3312	HSPA8	heat shock 70 kDa protein 8	**0,49**
NM_004346	836	CASP3	caspase 3, apoptosis-related cysteine peptidase	**0,45**
NM_003029	6464	SHC1	SHC (Src homology 2 domain containing) transforming protein 1	**0,45**
NM_005658	7185	TRAF1	TNF receptor-associated factor 1	**0,44**
NM_032904	5781	PTPN11	protein tyrosine phosphatase, non-receptor type 11 (Noonan syndrome 1)	**0,42**
NM_005544	3667	IRS1	insulin receptor substrate 1	**0,25**
NM_003246	7057	THBS1	thrombospondin 1	**0,20**
NM_005243	2130	EWSR1	Ewing sarcoma breakpoint region 1	**-0,46**
NM_003177	6850	SYK	spleen tyrosine kinase	**-0,48**
NM_021105	5359	PLSCR1	phospholipid scramblase 1	**-0,48**
NM_001753	857	CAV1	caveolin 1, caveolae protein, 22 kDa	**-0,50**
AF130085	1499	CTNNB1	catenin (cadherin-associated protein), beta 1, 88 kDa	**-0,56**
NM_015400	4088	SMAD3	SMAD, mothers against DPP homolog 3 (Drosophila)	**-0,62**
NM_001101	60	ACTB	actin, beta	**-0,63**
NM_007315	6772	STAT1	signal transducer and activator of transcription 1, 91 kDa	**-0,94**
AK024192	11030	RBPMS	RNA binding protein with multiple splicing	**-0,97**
NM_000633	596	BCL2	B-cell CLL/lymphoma 2	**-1,41**

Columns show: the GenBank accession number, the EntrezGene ID, the Gene symbol, the gene's description and the geometric mean of the log_2 _ratio given in Additional file 1, column N, indicating the level of gene expression.

The hubs could be categorized in different ways, e.g. on the basis of a specific GO term being over-represented in the protein group formed by the hub and the interacting proteins. Some nodes, such as SMAD3, CDKN1A and CCND1, interacting with proteins that belong to the same cellular compartment, could be defined as "party hubs" [12]. Other hubs, such as TGFBR1 and PXN, interact with proteins that have a different cellular localization and could be defined as "date hubs". Moreover, TGFBR1 and PXN tend to interact with proteins that act as bridges with other hubs, thus becoming more interconnected than other proteins. As shown by Han et al. [12] in yeast, party and date hubs may have different functions. In particular, date hubs seem to take part in a wide range of integrated connections required for the global organization of biological modules in the whole proteome network.

To validate the microarray data, RT/PCR was performed for six selected genes belonging to the most relevant GO categories identified by our analysis. The RNA samples used for RT/PCR analysis were those used for the microarrays. The up-regulation of tenascin (TNC), fibronectin 1 (FN1), matrix metalloproteinase 2 (MMP2) and connective tissue growth factor (CTGF) and the down-regulation of SMAD3 and collagen IV were confirmed by the RT/PCR experiments. All these genes proved to be similarly regulated by TGFβ1 in all three stimulation experiments.

## Discussion

We used the global gene expression profile approach to identify context-dependent markers of the EMT obtained from the long-term TGFβ1 treatment of HUTEC in primary culture. Based on our previous data, we had speculated that the context-dependent EMT process we obtained was a dedifferentiating event. One of the aims of the present study was to further substantiate this hypothesis.

Various studies have shown that genes with a similar expression pattern frequently display common functions and form networks of interacting proteins [13]. Assuming that the genes identified in our experiments belong to the TGFβ1-regulated pathways, we searched for interactions between the proteins encoded by the differentially expressed genes given in Additional file 1. We reasoned that microarray analysis might identify only a part of the complex TGFβ1 network, due to other effects such as post-transcriptional regulation, so we used protein-protein interaction data to identify proteins interacting with those encoded by differentially expressed genes. We obtained a single connected component (interactome) consisting of 2630 proteins and containing 449 differentially expressed proteins that interact directly or with undifferentially expressed proteins. This analysis is extremely useful not only for detecting the network of interacting proteins that respond to TGFβ1, but also for identifying the network hubs, i.e. proteins with a high degree of connectivity that could have a crucial role in response to TGFβ1. We identified 27 hubs with more than 29 edges incident on them and encoded by genes found differentially expressed in our experiments. Of the three hubs identified as having more than 12 interactions with differentially expressed genes, SMAD3 was classified as a party hub while TGFBR1 and PXN were considered as date hubs. As shown by Han et al. [12] in yeast, party and date hubs might have markedly different global properties in the interactome network. In accordance with this model, TGFBR1 and PXN, which interact with proteins that have a different cellular localization, might represent global or "high level" connectors between different biological modules and SMAD3, which functions within a module, works at a "lower level" in the organization of the proteome.

**Figure 5:**
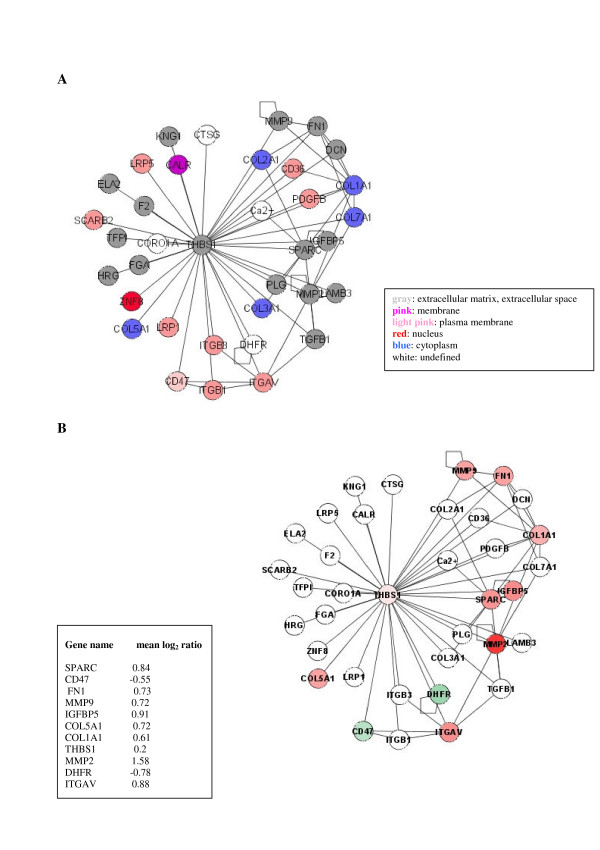
**Topological connections of the date hub THBS1**. Thrombospondin 1 (THBS1) links directly 35 proteins in the TGFβ1 interactome: A) the topological connections are shown; B) the proteins encoded by differentially expressed genes are indicated and colored respectively in red and green using a color gradient reflecting the mean log_2 _ratio reported in the square.

Among hubs, thrombospondin 1 (THBS1) – the principal activator of the TGFβ1 peptide [14] – emerges because it links a wide range of matrix proteins mediating their interaction with cell-surface receptors. Its central role is confirmed by the 35 links connecting this protein to the other proteins in the interactome map. It could be considered as a date hub in the TGFβ1 interactome, according to the definition given by Han et al. [12]. In Figure 5, the topological connections of THBS1 are magnified (A) and the differentially expressed genes interacting with it are indicated (B). Moreover, THBS1 is the gene that has the most significant correlation with TGFβ1 dosage (Table 1).

THBS1, PLS3 and ITGAV are known to have a pivotal role, both in cytoskeleton remodeling and in de novo extracellular matrix synthesis, two of the most relevant processes in EMT. The importance of these processes is highlighted by the considerable number of differentially expressed genes in the "extracellular matrix" and "cytoskeleton" GO categories (Figure 2).

In the "extracellular matrix (EMC)" category, the different regulation of the various collagen types emphasizes the transition from an epithelial-like to a mesenchymal ECM. The up-regulation of TNC and FN1, one of the hubs of the TGFβ1 interactome, confirmed the switch to a mesenchymal ECM. The laminin α chain was found differentially down-regulated in our model, as in other EMT models [5], while the β1 chain is up-regulated, as occurs in the precondensed mesenchyma [15], suggesting that an embryonal mesenchymal ECM is being synthesized. This suggestion is reinforced by the types of integrins up-regulated during EMT. In fact, the "ECM receptor interaction" of the KEGG pathways (Additional file 2) reveals not only the up-regulation of integrins generating the network for mesenchymal cell adhesion (ITGA5 and ITGAV) [16], but also – and above all – the strong over-expression of ITGA11, which constitutes the receptor for the interstitial collagens involved in cell migration and collagen reorganization on mesenchymal cells during development [17].

The "cytoskeleton" category is characterized by a large number of up-regulated genes, some of which have a crucial role in cytoskeleton remodeling. It is worth noting that four hubs of the TGFβ1 network, i.e. filamin A, alpha (FLNA), alpha 1 actin (ACTA1), paxillin (PXN) and beta actin (ACTB), belong to this category.

"Morphogenesis" is the GO category with the largest number of up-regulated genes, as shown clearly in Figure 3. The significance score assigned by the GOMiner software is not very high, however, because this category is one of the most represented in the microarray chip. It should nonetheless be noted that some of the genes belonging to this category are among the top ten up-regulated differentially expressed genes (Additional file 1). Stimulated by TGFβ1, HUTEC seem to reactivate the developmental processes: this may point to a sort of stemness of tubular cells that enables them to dedifferentiate when stimulated (kidney repair and maintenance), but also to reawaken a silenced embryogenetic program. Sox 11, GADD45B, N-cadherin (CDH2), Activin A (INHBA), CTGF, FGF1/5, Angiopoietin (ANGPTL4), natriuretic peptide precursor B (NPPB), calcitonin receptor (CALCR) and caldesmon 1 (CALD1) are the most over-expressed genes belonging to the "morphogenesis" category. That tubular cells have the ability to reactivate an embryogenetic program has been demonstrated very recently by Kitamura et al. [18] and Maeshima et al. [19], who identified and isolated cells of the S2 segment with a potential tubulogenic ability and a capacity for integration in the developing kidney. Our results are in line with their findings.

CD133+ cells have been found to have stem cell potential in the adult kidney [20]. Hypothesizing that CD133 up-regulation should occur if a stemness property is activated by TGFβ1, we looked specifically for prominin 1 (CD133) mRNA activation in our EMT model. Surprisingly, we found CD133 down-regulated, indicating that CD133 is expressed in control conditions. This finding supports the view taken by Florek et al. [21], who showed that prominin 1 transcript and the alphahE2 epitope immunoreactivity of CD133 (obtained using a novel antibody instead of AC133) occur in several adult tissues and in the proximal tubular cells of the adult kidney in particular.

Signaling members of the Wnt (WNT5B) and FGF (1/5) families and transcription factors such as Sox 11, known to have a crucial role in nephrogenesis and cell fate determination during kidney development [22,23], were found up-regulated. The involvement of the Wnt pathway was also confirmed by KEGG analysis.

The non-canonical Wnt signaling pathway centered on WNT5/Ca^2+ ^[24,25] seems to be activated through Nemo-like kinase (NLK) in our EMT model and to antagonize the canonical beta-catenin Wnt signaling (Additional file 2). In fact, we observed the down-regulation of both wingless-type MMTV integration site family, member 2B (WNT2B) and catenin, beta 1 (CTNNB1) and the up-regulation of both WNT5B and the calcium signaling pathway (see KEGG pathways). This effect seems to be reinforced by the up-regulation of dapper, antagonist of beta-catenin, homolog 1 (DACT1), which is a known beta-catenin Wnt signal inhibitor. It has been reported that inhibiting the beta-catenin system strongly inhibited TGFβ1-induced αSMA expression in tubular cells [26]. Our data confirm this finding, since no αSMA expression was triggered by TGFβ1 in our EMT model [6].

The non-canonical Wnt pathway, which includes planar cell polarity – an important process in embryonal axis development involving cytoskeletal polarity, as well as in the calcium pathway regulating cell adhesion [24]-, was thus found up-regulated in our EMT model, reinforcing the idea that an embryological program is awakened. Very recently, Osafune et al. [25] reported that the capacity of renal progenitor cells of the metanephric mesenchyme to form colonies in vitro and undergo mesenchymal-epithelial transition is positively regulated by planar cell polarity pathways downstream from Wnt.

Although the canonical Wnt signaling was repressed, the final effector of the pathway – and one of the most important – Cyclin D1 (CCND1) was up-regulated, whereas Cyclin B2 (CCNB2) – which is assumed to bind to TGFβ R2 and thus play a key part in TGFβ-mediated cell cycle control – was down-regulated.

Apoptosis and EMT are two distinct and opposite signal modules for TGFβ1-downstream effects. There is growing evidence that SMAD3 is an important signaling anchor for the apoptotic network for TGFβ1 too. In particular, the loss of SMAD3 function due to a decrease in its expression might be a requirement for epithelial cells to survive in the presence of prolonged TGFβ1 stimulation [27]. This was also confirmed in our EMT model: visual inspection of the TGFβ-SMAD KEGG pathway (Additional file 2) reveals what we demonstrated previously using RT/PCR analysis [6], i.e. that Smad signaling was down-regulated. Despite the number of up-regulated inducers (TGFβ1, INHBA), the key transducers are all down-regulated, as are the Id genes (the effectors of cell differentiation). The ID2B gene was specifically down-regulated. Mad expression and ID2 down-regulation are important events in the TGFβ1 cytostatic program in epithelial cells and ID2 suppression by TGFβ1 is essential for EMT to occur [28,29].

**Figure 6:**
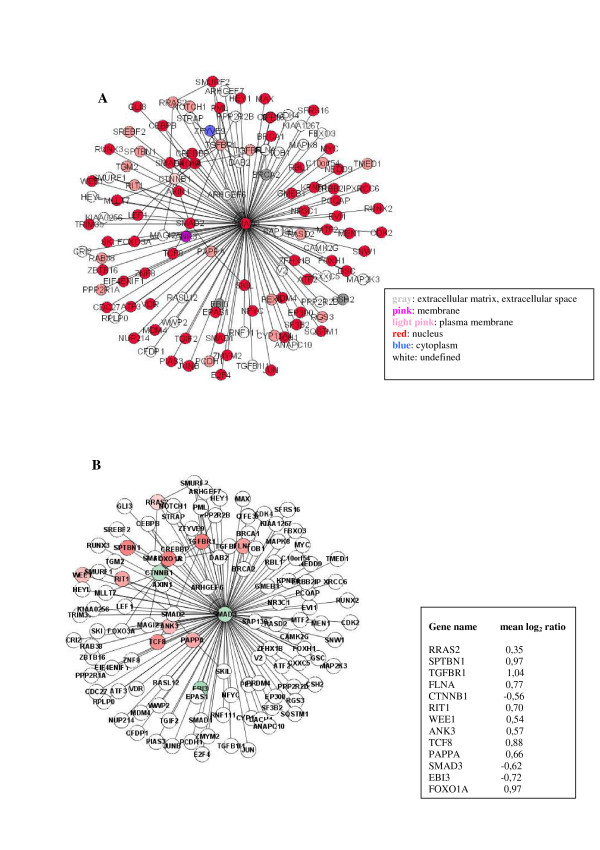
**Topological connections of the party hub SMAD3**. SMAD3 links directly 119 proteins in the TGFβ1 interactome: A) the topological connections are shown; B) the proteins encoded by differentially expressed genes are indicated and colored respectively in red and green using a color gradient reflecting the mean log_2 _ratio reported in the square.

The central role of SMAD3 is also demonstrated by its position in the TGFβ1 network: it is one of the hubs, most likely a date hub since it works within a single module (Figure 6), at a low level of network organization. This might explain why apoptosis seems to be induced in our model, despite SMAD2 and SMAD3 down-regulation. In fact, a visual inspection of the KEGG apoptosis pathway clearly shows the up-regulation of caspase 3 (CASP3), a known inducer of cell death, and the under-expression of BCL2 and BIRC3 (IAP gene in the KEGG map), which counteract this action.

On the other hand, the up-regulation of several genes implicated in the cell cycle pathway (Additional file 2), such as CCND1, GADD45, YWHAG (tyrosine 3-monooxygenase/tryptophan 5-monooxygenase activation protein), indicates that cells are entering the cell cycle. In this pathway, however, the up-regulation of wee1 tyrosine kinase (WEE1), one of the genes strictly regulated by TGFβ1, seems to indicate a sort of actual control of cell proliferation, so both apoptosis and cell cycle entry seem to be strictly controlled in the EMT process. Indeed, neither apoptosis nor proliferation were seen in our model by immunocytochemistry [6]. It is tempting to speculate that a concerted and strictly controlled action between signals for cell death and proliferation might be taking place in cells after long-term TGFβ1 exposure, miming the developmental process in which morphogens such as TGFβ1 act not as positive regulators of cell differentiation but as key regulators of cell survival [30]. It is worth noting that key proteins of the Wnt signaling and apoptosis/cell cycle control pathways (i.e. CTNNB1, CCND1, CASP3, BCL2) were found to constitute some of the hub proteins of the TGFβ1 network.

Finally, KEGG analysis of microarray data highlighted that RAS/MAPK signaling was the principal downstream effector of chronic TGFβ1 stimulation in our EMT model, confirming the suggestions advanced by other authors [5,8], i.e. that both Smad-dependent and Smad-independent signaling cascades are activated by TGFβ1 and that they regulate mesenchymal transition in a context- and cell-dependent manner. The MAPK signaling pathway has an important role in connecting the signal triggered by TGFβ1 to important downstream processes such as apoptosis/proliferation and the Wnt cascade [5]. Our results confirm reports from other authors on the role of this signaling in other EMT processes [31-33].

## Conclusion

Understanding how mesenchymal cells arise from epithelial cells could have a strong impact in unveiling the mechanism behind fibrosis and cancer progression. Moreover, it might reveal mechanisms of epithelial cell plasticity underlying kidney regeneration and repair.

In the kidney, tissue regeneration and repair occur through three, not mutually exclusive, cellular and molecular mechanisms: differentiation of the somatic stem cells, recruitment of circulating stem cells and, more importantly, proliferation/dedifferentiation of mature cells. Dedifferentiation seems to represent a critical step for the recovery of tubular integrity and precedes the reconstitution of a well-differentiated morphology. Understanding the cellular and molecular events involved in renal tubule regeneration is indispensable to design cell-based and other therapeutic strategies in order to potentiate this innate capacity. EMT is now considered a part of tubular cell plasticity.

The purpose of our study was to substantiate our initial hypothesis that the process of EMT induced by TGFβ1 chronic exposure in HUTEC is a dedifferentiating process. Our earlier results suggested that this might be so and our present findings support that impression. In fact: 1) the principal functional category involved in the EMT process concerns morphogenesis and development; 2) the most up-regulated genes belong to this category; and, finally, 3) some intracellular pathways are involved, whose engagement during kidney development and nephrogenesis is well known. Our results thus suggest that the recapitulation of embryological programs is an integral part of the EMT process on long-term exposure to TGFβ1 and that tubular cells may have the capacity, under appropriate environmental cues, to redifferentiate not only back to an epithelial type but also towards another cell type, i.e. myofibroblasts or endothelial cells. Judging from our data, moreover, TGFβ1 seems (in the context of our experiments) to act as a morphogen regulating cell survival by means of strictly balanced signals for cell death and proliferation.

Finally, our findings are the first to show that genes involved in the TGFβ1-driven EMT process are highly interconnected and topologically related in the human interactome map. They generate a single scale-free network whose hub proteins were found differentially expressed, pointing to a crucial role for them in the EMT process. The main role of one of them, thrombospondin 1, emerges from its high degree of regulation by TGFβ1 and from the 35 links connecting this protein to the other proteins in the interactome map.

## Methods

### Cell cultures

Primary HUTEC cultures were established as explained elsewhere [6]. Cells at passage 1 were used for TGFβ1 stimulation experiments, designed to monitor the effect of TGFβ1 at both phenotypic and molecular levels simultaneously [6]. Cells were seeded at subconfluence and incubated at 37°C in a 5% CO_2 _atmosphere for 24 hrs under quiescent conditions (1% FCS in RPM1 1640) in 6-well plastic or collagen I-coated plates for RNA extraction. Cells were cultured for 4 days in the presence of 1, 5, 10, 50 ng/ml human TGFβ1 (Prepro Tech EC, London, UK). Stimulation experiments were conducted in triplicate and morphological, immunocytochemical and molecular analyses were performed. Control conditions were represented by cells maintained for 4 days in 1% serum without TGFβ1.

### RNA extraction and quality control

Total RNA was extracted using RNAzolB (BIOTEX, Houston, USA) according to the protocol.

The Agilent 2100 Bioanalyzer (Agilent Technologies, PaloAlto, USA) was used to assess RNA integrity. RNA was quantified with the UV/VIS Spectrometer (Lambda 2S PerkinElmer).

### Microarray platform

Microarray expression was analyzed using the Operon 70 mer oligos collection (Human Version 2.0) containing 21,329 oligonucleotides spotted in duplicate (GPL2136136 record in the GEO database) on MICROMAX glass slides – SuperChip I provided by PerkinElmer Life Sciences Inc. (Boston, USA).

Oligos were printed using the Biorobotics Microgrid II spotter; spots were spaced 115 μm and microarrays consisted of 48 subarrays.

### RNA amplification and labeling

1 μg of total RNA was amplified using the Amino Allyl MessageAmp™ aRNA Kit (Ambion, Austin, USA). Five μg of amplified RNA (aRNA) were labeled with Cy3/Cy5 fluorophores using CyDye Post Labeling Reactive Dyes (Amersham Biosciences, Piscataway, USA).

Following purification, dye incorporation was quantified by spectrophotometric analysis.

### Hybridization

Approximately 2.5 μg of aRNA labeled with 100 pmoles of fluorophore were used for each hybridization.

Labeled aRNA was precipitated using NH_4_Ac and EtOH following standard protocols and resuspended in hybridization buffer (5× SSC; 0.1% SDS; 40 ng/μl SS-DNA; 25% formamide).

Microarray slides were pre-hybridized in GeneMachines™ chambers (Genomic Solutions, Cambs., UK) for 2 h at 48°C with 70 μl of pre-hybridization buffer (5× SSC; 0.1% SDS; 40 ng/μl SS-DNA; 1× Denhardt's solution) using a coverslip.

Slides were washed with water and dried with compressed air.

Hybridization was carried out using Hybridization Station ArrayBooster™ (Advalytix, Brunnthal, Germany) at 48°C for 12 h.

Microarrays were washed with 1× SSC plus 0.2% SDS for 4 minutes, 0.1× SSC plus 0.2% SDS for 4 minutes, twice with 0.2× SSC for 4 minutes, and twice with 0.1× SSC for 3 minutes.

### Microarray scanning and image analysis

Microarrays were scanned using ScanArray Lite (PerkinElmer™). Images were analyzed using ScanArray Express (PerkinElmer™).

### Statistical analysis

The following procedure was used to remove spots with a low fluorescence intensity or high variability between replicates.

#### 1) "Intensity-dependent calculation of standard Z-score"

spots with a median fluorescence pixel intensity below 700 (calculated considering negative control intensity) on both Cy3 and Cy5 channels were filtered out; those with a median fluorescence pixel intensity of zero or less in only one channel were set to 100 to prevent their elimination during normalization. Files were saved in "tav" format to make them suitable for reading with MIDAS software [34] and normalization was done using the LOWESS method.

Two different procedures (named A and B) were used to eliminate outliers, as follows.

(A) Based on the method suggested by Yang et al. [35], we calculated R1 = (CH1 intensity/CH2 intensity) and R2 = (CH1 intensity/CH2 intensity) values for the two replicates of the same gene on the microarray and log_2_(R1/R2). We indicated the two replicates of the spot as R1 and R2. Then we calculated the mean and SD (standard deviation) for the log_2_(R1/R2) values of all microarray spots. Those with a log_2 _ratio higher than |3 SD| were rejected due to replicate inconsistency.

The geometric mean for the two replicates of the remaining genes was calculated and the output files were saved in "tav" format.

(B) The "tav" file for each microarray experiment was normalized using MIDAS software and geometric mean values underwent SLICE data analysis, considering only those where |log_2_(CH1 intensity/CH2 intensity)|≥1.5 SD.

Each experiment was performed in duplicate using a dye swap procedure and only the genes that independently complied with these filters on both replicates were considered.

#### 2) "SAM analysis"

data were also analyzed using SAM software, but intra-array replicates were not averaged, intensity fluorescence filtering was as described previously and normalization was done using the LOWESS procedure. The four replicates (two intra-array and two deriving from the dye-swap analysis) were t-tested using the SAM software and considering the lowest False Discovery Rate.

In all, 1203 spots were considered at this stage: 589 of them satisfied both statistical procedures 1) and 2) in at least one of the seven experiments (collagen 5–10–50, plastic 5–10–50 and collagen vs. plastic) and 614 spots were identified by the SAM software alone. The geometric mean was calculated from the four replicates of each gene and is given in Additional file 1 (columns G-L). Some genes, especially those identified only by the SAM software and in the "50 ng of TGFβ1" experiments, had a low (<0.65) log_2 _ratio and were filtered out. We finally obtained 993 differentially expressed spots, corresponding to 977 genes (some oligos refer to the same gene), given in Additional file 1. This table consists of two parts: spots 1 to 554 are the most significant because they satisfy both statistical procedures 1) and 2); genes from 555 to 993 are less significant because they only passed the SAM analysis.

We also used the "quantitative response" option of the SAM software to check for the presence of genes whose expression is regulated in a dose-dependent manner (Table 1).

### Validation of microarray data

#### RT/PCR-Real Time PCR

six differentially expressed genes belonging to the different GO categories found involved in the EMT process, i.e. TNC, FN1, collagen IV, MMP2, SMAD3 and CTGF, were analyzed. Quantitative comparative RT/PCR and Real Time RT/PCR were performed, as reported elsewhere [6].

#### Immunocytochemistry

immunocytochemistry was conducted, using antibodies against αSMA, cytokeratin 8–18, vimentin, collagen III, Ki67 and E-cadherin, as described in [6].

### Annotation checking procedure

The annotation of the Human Version 2.0 microarray platform (OPERON) was out of date, so we double-checked it using the MatchMiner software [36], loading the GenBank accession numbers and recovering the EntrezGene ID and the gene symbol (only for the genes in Additional file 1). If the GenBank clone had been withdrawn, we performed a BLAST search with the oligo sequence on the UCSC Genome Browser [37] and we updated the GenBank ID. This search was also conducted for the RefSeq sequences with multiple associations. Oligos matching more than one gene were rejected. To verify gene symbols and gene descriptions, we downloaded the "gene_info.gz" file from the NCBI ftp site [38].

### Functional analysis of microarray data

Geometric mean expression values of the 993 spots identified were obtained for each of the seven experiments and these data were clustered using TMEV software [34].

Functional categories were identified with the GoMiner software [39] using the Gene Ontology (GO) annotation [40]. We selected a p value threshold of 0.02 for Biological Process categories and 0.05 for the Cellular Component.

To identify KEGG pathways involved in the TGFβ1 response we colored up- or down-regulated genes with the tool available on the KEGG site [41,42]. Differentially expressed genes that only passed the SAM analysis were colored orange (up-regulated) or light blue (down-regulated); genes that passed both statistical analyses (see Methods) were colored red (up-regulated) or blue (down-regulated).

### Mapping the differentially expressed genes in the human interactome

We used data from the literature on protein-protein interactions to verify whether the genes in Additional file 1 are independent or tend to be associated with one another in one or more clusters. We downloaded the protein-protein interactions from three different sources, i.e. (A) the NCBI ftp site [43] (interactions.gz), from which we selected the interactions identified in Homo sapiens and used a Perl script to assign the official symbol to the known proteins; (B) the BioGRID database [44]; and (C) the Biomolecular Interaction Network Database (BIND) [45]. Each data set was loaded separately in the Cytoscape software [46] and three distinct "global" protein-protein interaction networks were generated. For each global network we mapped the genes identified in our microarray experiments (Additional file 1) and selected proteins that interact with them directly, generating a small group of sub-networks. We selected only the three largest sub-networks and they were merged into a single subnetwork using the "union" function in Cytoscape. The network we obtained is composed of 2630 nodes and 4183 edges. We used the "filter option" in Cytoscape to select the nodes interacting with more than 29 proteins. These nodes were called hubs. Gene Ontology analysis was performed on the network using BINGO software [47], while network topological statistics were obtained using tYNA software [10]. The interactome was also deposited in the tYNA database [48] (TGFbeta-network-231106). Microarray experiments were loaded into ArrayExpress with accession number E-MEXP-566 according to the MIAME rules [49].

## Authors' contributions

SC and SP contributed equally to this work: they took part in the microarray experiments, performed the statistical and data integration analyses and drafted the manuscript.

RT participated in the interpretation of the data.

LC conducted the microarray experiments.

MC carried out in vitro experiments and RNA purification.

DDP carried out immunocytochemical analyses.

ADA approved the final version for publication.

GV participated in the design of the study and coordinated the statistical analyses.

FA conceived the study, took part in its design and coordination, and helped draft the manuscript.

All Authors have read and approved the final version of the manuscript.

## Supplementary Material

Additional file 1 **List of differentially expressed genes**. Genes numbered from 1 to 554 (column A) passed both statistical analyses (SAM and "intensity-dependent calculation of standard Z-score", see Methods), those numbered from 555 to 993 passed only the SAM analysis. The other columns show: (B) the OPERON oligo ID in the OMAD database [50]; (C) the EntrezGene ID; (D) the Gene Symbol; (E) the description of the gene ; (F) genes belonging to the cluster of interacting genes in Figure 3, marked "1"; (G-L) genes up-regulated in cells treated with TGFβ1 have log^2 ^values higher than 0, genes down-regulated have values lower than 0 (values that passed the statistical analysis are in bold); (M) genes up-regulated in the plastic substrate by comparison with the collagen type I have values higher than 0, genes down-regulated have values lower than 0; (N) geometric means of the experiments in columns (G-L). Click here for file

Additional file 2 **KEGG analysis of differentially expressed genes**. 267 genes in Additional file 1 with an EntrezGene ID were placed in the KEGG maps using the "color genes" option in the KEGG database [41]. Differentially expressed genes that only passed the SAM analysis were colored orange (up-regulated) and light-blue (down-regulated); genes that passed both statistical analyses (see Methods) were colored red (up-regulated) and blue (down-regulated). Click here for file

## References

[B1] Anglani F, Forino M, Del Prete D, Tosetto E, Torregrossa R, D'Angelo A (2004). In search of adult renal stem cells. J Cell Mol Med.

[B2] Stahl PJ, Felsen D (2001). Transforming growth factor-beta, basement membrane, and epithelial-mesenchymal transdifferentiation: implications for fibrosis in kidney disease. Am J Pathol.

[B3] Iwano M, Plieth D, Danoff TM, Xue C, Okada H, Neilson EG (2002). Evidence that fibroblasts derive from epithelium during tissue fibrosis. J Clin Invest.

[B4] Massague J, Wotton D (2000). Transcriptional control by the TGF-beta/Smad signaling system. EMBO J.

[B5] Zavadil J, Bottinger EP (2005). TGF-beta and epithelial-to-mesenchymal transitions. Oncogene.

[B6] Forino M, Torregrossa R, Ceol M, Murer L, Vella MD, Prete DD, D'Angelo A, Anglani F (2006). TGFbeta1 induces epithelial-mesenchymal transition, but not myofibroblast transdifferentiation of human kidney tubular epithelial cells in primary culture. Int J Exp Pathol.

[B7] Zavadil J, Bitzer M, Liang D, Yang YC, Massimi A, Kneitz S, Piek E, Bottinger EP (2001). Genetic programs of epithelial cell plasticity directed by transforming growth factor-beta. Proc Natl Acad Sci USA.

[B8] Derynck R, Zhang YE (2003). Smad-dependent and Smad-independent pathways in TGF-beta family signaling. Nature.

[B9] Bottinger EP, Bitzer M (2002). TGF-beta signaling in renal disease. J Am Soc Nephrol.

[B10] Yip KY, Yu H, Kim PM, Schultz M, Gerstein M (2006). The tYNA platform for comparative interactomics: a web tool for managing, comparing and mining multiple networks. Bioinformatics.

[B11] Sampogna RV, Nigam SK (2004). Implications of gene networks for understanding resilience and vulnerability in the kidney branching program. Physiology (Bethesda).

[B12] Han JD, Bertin N, Hao T, Goldberg DS, Berriz GF, Zhang LV, Dupuy D, Walhout AJ, Cusick ME, Roth FP, Vidal M (2004). Evidence for dynamically organized modularity in the yeast protein-protein interaction network. Nature.

[B13] Li S, Armstrong CM, Bertin N, Ge H, Milstein S, Boxem M, Vidalain PO, Han JD, Chesneau A, Hao T, Goldberg DS, Li N, Martinez M, Rual JF, Lamesch P, Xu L, Tewari M, Wong SL, Zhang LV, Berriz GF, Jacotot L, Vaglio P, Reboul J, Hirozane-Kishikawa T, Li Q, Gabel HW, Elewa A, Baumgartner B, Rose DJ, Yu H, Bosak S, Sequerra R, Fraser A, Mango SE, Saxton WM, Strome S, Van Den Heuvel S, Piano F, Vandenhaute J, Sardet C, Gerstein M, Doucette-Stamm L, Gunsalus KC, Harper JW, Cusick ME, Roth FP, Hill DE, Vidal M (2004). A map of the interactome network of the metazoan C. elegans. Science.

[B14] Daniel C, Wiede J, Krutzsch HC, Ribeiro SM, Roberts DD, Murphy-Ullrich JE, Hugo C (2004). Thrombospondin-1 is a major activator of TGF-beta in fibrotic renal disease in the rat in vivo. Kidney Int.

[B15] Horster MF, Braun GS, Huber SM (1999). Embryonic renal epithelia: induction, nephrogenesis, and cell differentiation. Physiol Rev.

[B16] Zoppi N, Gardella R, De Paepe A, Barlati S, Colombi M (2004). Human fibroblasts with mutations in COL5A1 and COL3A1 genes do not organize collagens and fibronectin in the extracellular matrix, down-regulate alpha2beta1 integrin, and recruit alphavbeta3 instead of alpha5beta1 integrin. J Biol Chem.

[B17] Tiger CF, Fougerousse F, Grundstrom G, Velling T, Gullberg D (2001). alpha11beta1 integrin is a receptor for interstitial collagens involved in cell migration and collagen reorganization on mesenchymal nonmuscle cells. Dev Biol.

[B18] Kitamura S, Yamasaki Y, Kinomura M, Sugaya T, Sugiyama H, Maeshima Y, Makino H (2005). Establishment and characterization of renal progenitor-like cells from S3 segment of nephron in rat adult kidney. FASEB J.

[B19] Maeshima A, Sakurai H, Nigam SK (2006). Adult kidney tubular cell population showing phenotypic plasticity, tubulogenic capacity, and integration capability into developing kidney. J Am Soc Nephrol.

[B20] Bussolati B, Bruno S, Grange C, Buttiglieri S, Deregibus MC, Cantino D, Camusi G (2005). Isolation of renal progenitor cells from adult human kidney. Am J Pathol.

[B21] Florek M, Haase M, Marzesco AM, Freund D, Ehninger G, Huttner WB, Corbeil D (2005). Prominin-1/CD133, a neural and hematopoietic stem cell marker, is expressed in adult human differentiated cells and certain types of kidney cancer. Cell Tissue Res.

[B22] Scheld A, Hastie ND (2000). Cross-talk in kidney development. Curr Opin Genet Dev.

[B23] Plisov SY, Ivanov SV, Yoshino K, Dove LF, Plisova TM, Higinbotham KG, Karavanova I, Lerman M, Perantoni AO (2000). Mesenchymal-epithelial transition in the developing metanephric kidney: gene expression study by differential display. Genesis.

[B24] Widelitz R (2005). Wnt signaling through canonical and non-canonical pathways: recent progress. Growth Factors.

[B25] Osafune K, Takasato M, Kispert A, Asashima M, Nishinakamura R (2006). Identification of multipotent progenitors in the embryonic mouse kidney by a   novel colony-forming assay. Development. 2006 Jan;133(1):151-61.. Development.

[B26] Masszi A, Fan L, Rosivall L, McCulloch CA, Rotstein OD, Mucsi I, Kapus A (2004). Integrity of cell-cell contacts is a critical regulator of TGF-beta 1-induced epithelial-to-myofibroblast transition : role for beta-catenin. Am J Pathol.

[B27] Nicolas FJ, Lehmann K, Warne PH, Hill CS, Downward J Kondo M (2003). Epithelial to mesenchymal transition in Madin-Darby canine kidney cells is accompanied by down-regulation of Smad3 expression, leading to resistance to transforming growth factor-beta-induced growth arrest. J Biol Chem.

[B28] Cubillo E, Tobiume K, Shirakihara T, Fukuda N, Suzuki H, Shimizu K, Takehara K, Cano A, Saitoh M, Miyazono K (2004). A role for Id in the regulation of TGF-beta-induced epithelial-mesenchymal transdifferentiation. Cell Death Differ.

[B29] Kowanetz M, Valcourt U, Bergstrom R, Heldin CH, Moustakas A (2004). Id2 and Id3 define the potency of cell proliferation and differentiation responses to transforming growth factor beta and bone morphogenetic protein. Mol Cell Biol.

[B30] Mehlen P, Mille F, Thibert C (2005). Morphogens and cell survival during development. J Neurobiol.

[B31] Bhowmick NA, Zent R, Ghiassi M, McDonnell M, Moses HL (2001). Integrin beta 1 signaling is necessary for transforming growth factor-beta activation of p38MAPK and epithelial plasticity. J Biol Chem.

[B32] Yang YC, Piek E, Zavadil J, Liang D, Xie D, Heyer J, Pavlidis P, Kucherlapati R, Roberts AB, Bottinger EP (2003). Hierarchical model of gene regulation by transforming growth factor beta. Proc Natl Acad Sci USA.

[B33] Xie L, Law BK, Chytil AM, Brown KA, Aakre ME, Moses HL (2004). Activation of the Erk pathway is required for TGF-beta1-induced EMT in vitro. Neoplasia.

[B34] Saeed AI, Sharov V, White J, Li J, Liang W, Bhagabati N, Braisted J, Klapa M, Currier T, Thiagarajan M, Sturn A, Snuffin M, Rezantsev A, Popov D, Ryltsov A, Kostukovich E, Borisovsky I, Liu Z, Vinsavich A, Trush V, Quackenbush J (2003). TM4: a free, open-source system for microarray data management and analysis. Biotechniques.

[B35] Yang IV, Chen E, Hasseman JP, Liang W, Frank BC, Wang S, Sharov V, Saeed AI, White J, Li J, Lee NH, Yeatman TJ, Quackenbush J (2002). Within the fold: assessing differential expression measures and reproducibility in microarray assays. Genome Biol.

[B36] Bussey KJ, Kane D, Sunshine M, Narasimhan S, Nishizuka S, Reinhold WC, Zeeberg B, Ajay W, Weinstein JN (2003). MatchMiner: a tool for batch navigation among gene and gene product identifiers. Genome Biol.

[B37] Kent WJ, Sugnet CW, Furey TS, Roskin KM, Pringle TH, Zahler AM, Haussler D (2002). The Human Genome Browser at UCSC. Genome Res.

[B38] NCBI ftp site "gene info" file. ftp://ftp.ncbi.nlm.nih.gov/gene/DATA/.

[B39] Zeeberg BR, Feng W, Wang G, Wang MD, Fojo AT, Sunshine M, Narasimhan S, Kane DW, Reinhold WC, Lababidi S, Bussey KJ, Riss J, Barrett JC, Weinstein JN (2003). GoMiner: A Resource for Biological Interpretation of Genomic and Proteomic Data. Genome Biol.

[B40] Ashburner M, Ball CA, Blake JA, Botstein D, Butler H, Cherry JM, Davis AP, Dolinski K, Dwight SS, Eppig JT, Harris MA, Hill DP, Issel-Tarver L, Kasarskis A, Lewis S, Matese JC, Richardson JE, Ringwald M, Rubin GM, Sherlock G (2000). Gene ontology: tool for the unification of biology. The Gene Ontology Consortium. Nat Genet.

[B41] Ogata H, Goto S, Sato K, Fujibuchi W, Bono H, Kanehisa M (1999). KEGG: Kyoto Encyclopedia of Genes and Genomes. Nucleic Acids Res.

[B42] Color Objects in KEGG Pathways. http://www.genome.jp/kegg/tool/color_pathway.html.

[B43] NCBI ftp site "interactions" file. ftp://ftp.ncbi.nlm.nih.gov/gene/GeneRIF/.

[B44] The BioGRID File download. http://www.thebiogrid.org/downloads.php.

[B45] Biomolecular Interaction Network Database. http://bond.unleashedinformatics.com/.

[B46] Shannon P, Markiel A, Ozier O, Baliga NS, Wang JT, Ramage D, Amin N, Schwikowski B, Ideker T (2003). Cytoscape: a software environment for integrated models of biomolecular interaction networks. Genome Res.

[B47] Maere S, Heymans K, Kuiper M (2005). BiNGO: a Cytoscape plugin to assess overrepresentation of gene ontology categories in biological networks. Bioinformatics.

[B48] tYNA. http://tyna.gersteinlab.org/tyna/.

[B49] ArrayExpress. http://www.ebi.ac.uk/arrayexpress/.

[B50] OPERON. http://www.operon.com/arrays/omad.php?.

