# Supplementary figures and images for: Genes involved in TGFβ1-driven epithelial-mesenchymal transition of renal epithelial cells are topologically related in the human interactome map
Source: BMC Genomics. 2007 Oct 22;8:383. doi: 10.1186/1471-2164-8-383 (PMC2174485; doi:10.1186/1471-2164-8-383)

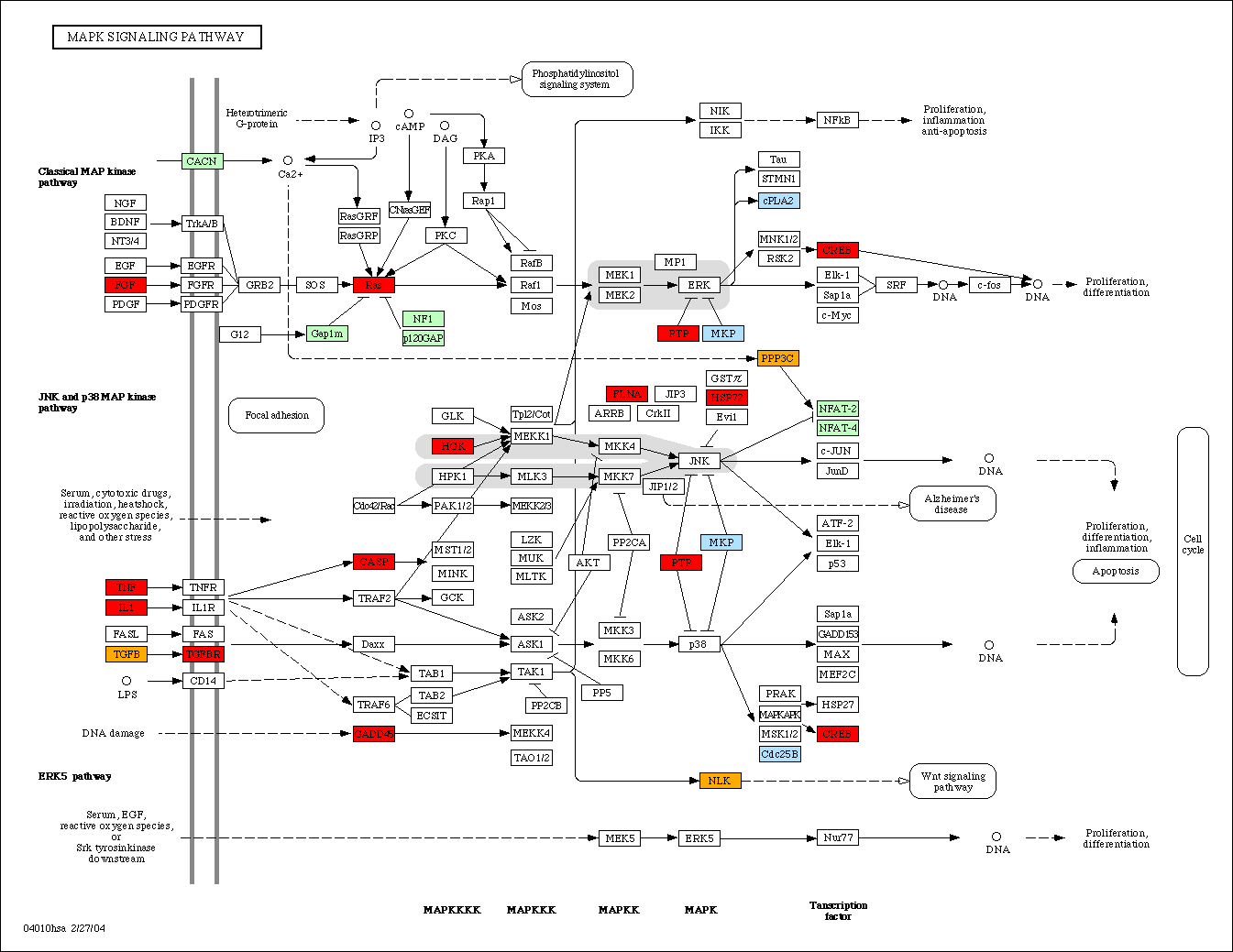


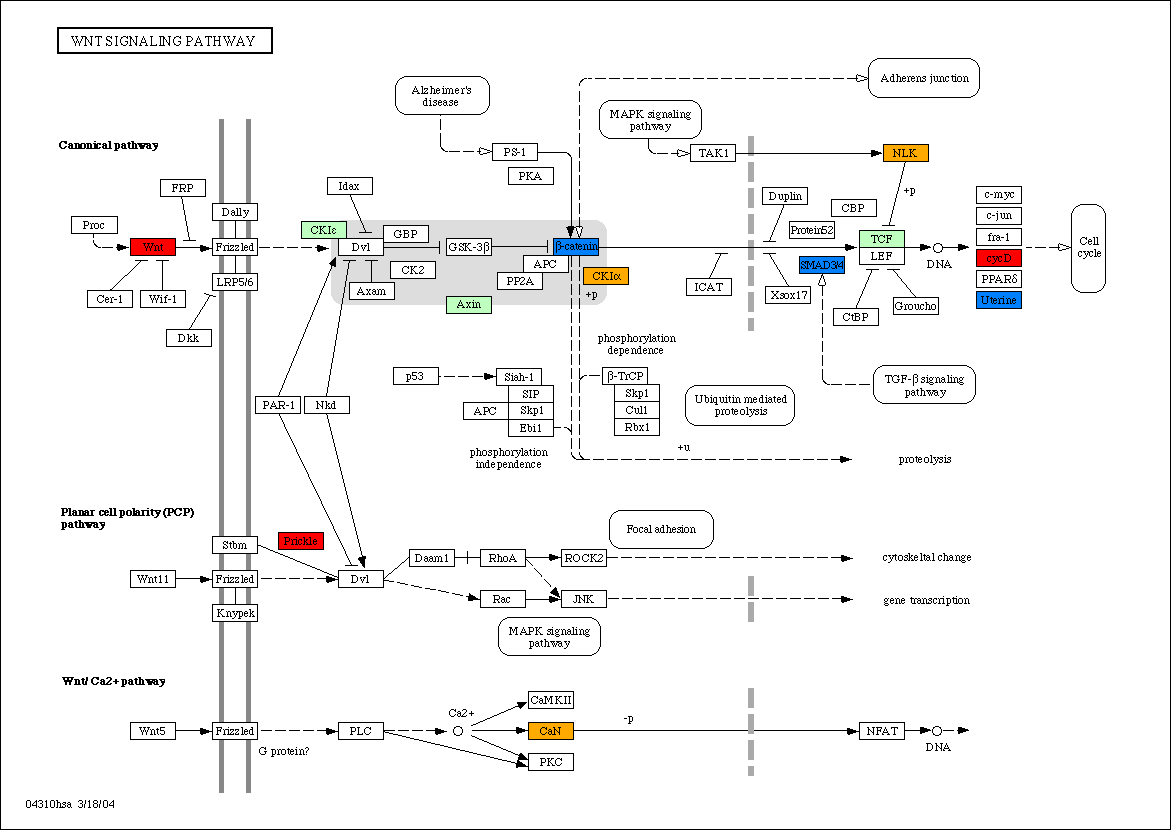


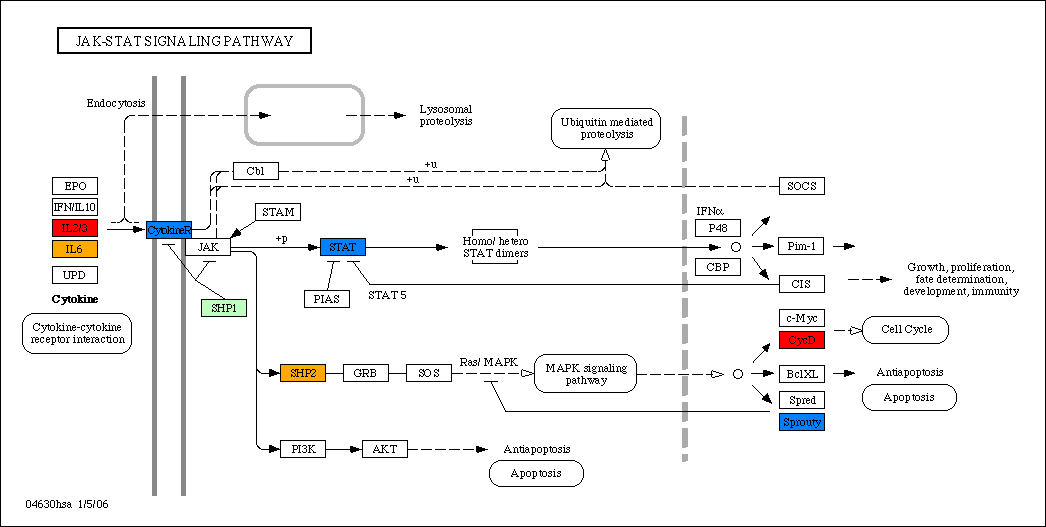


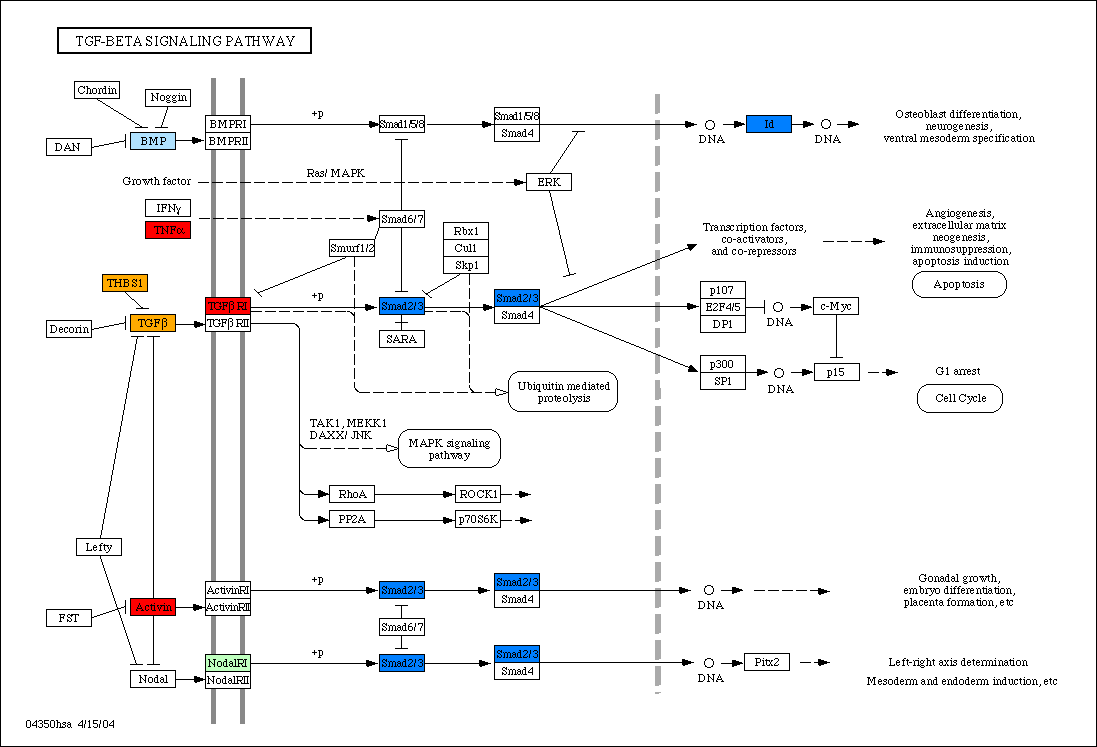


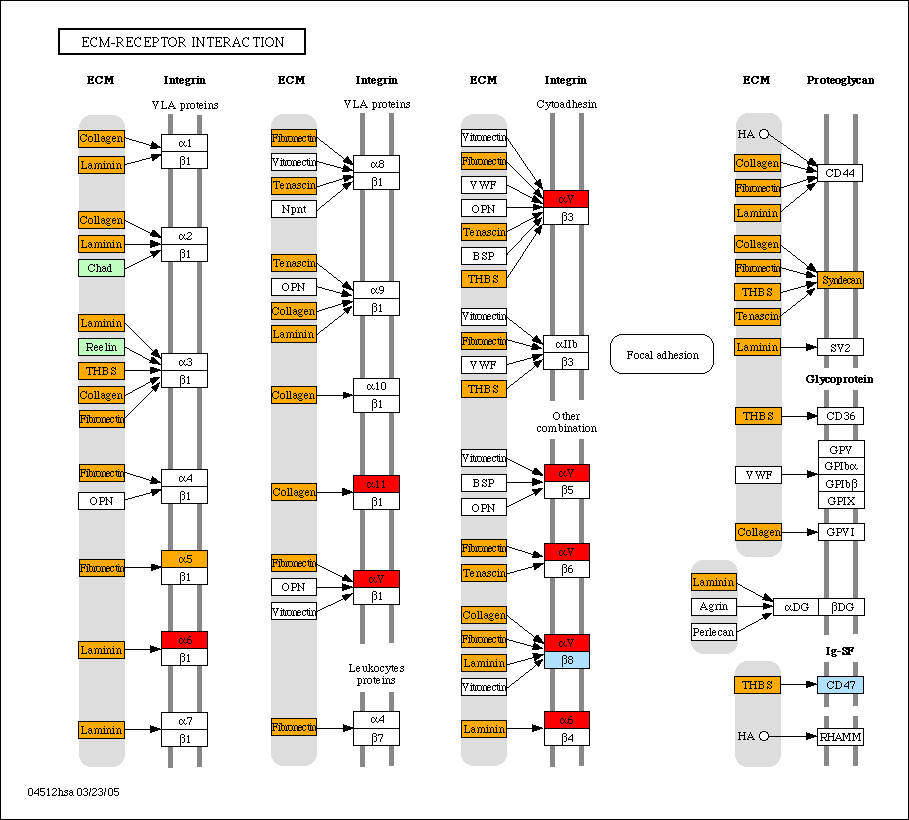


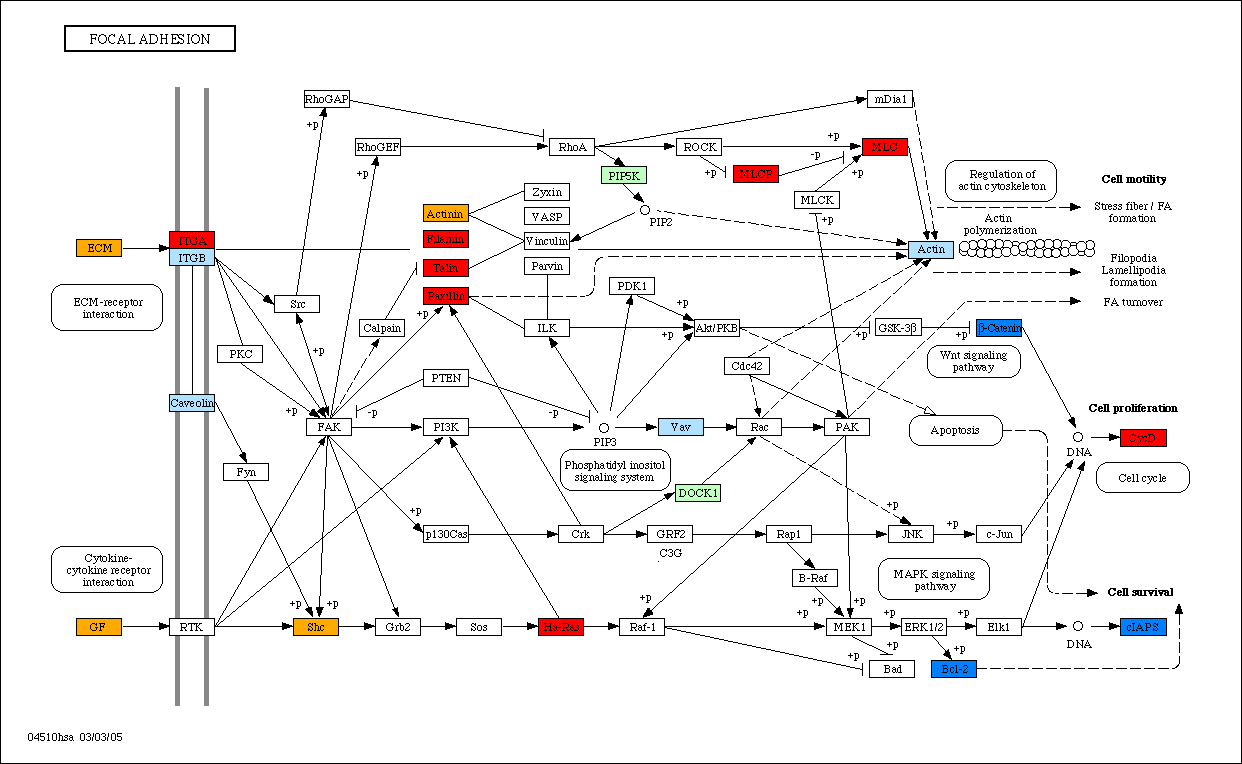


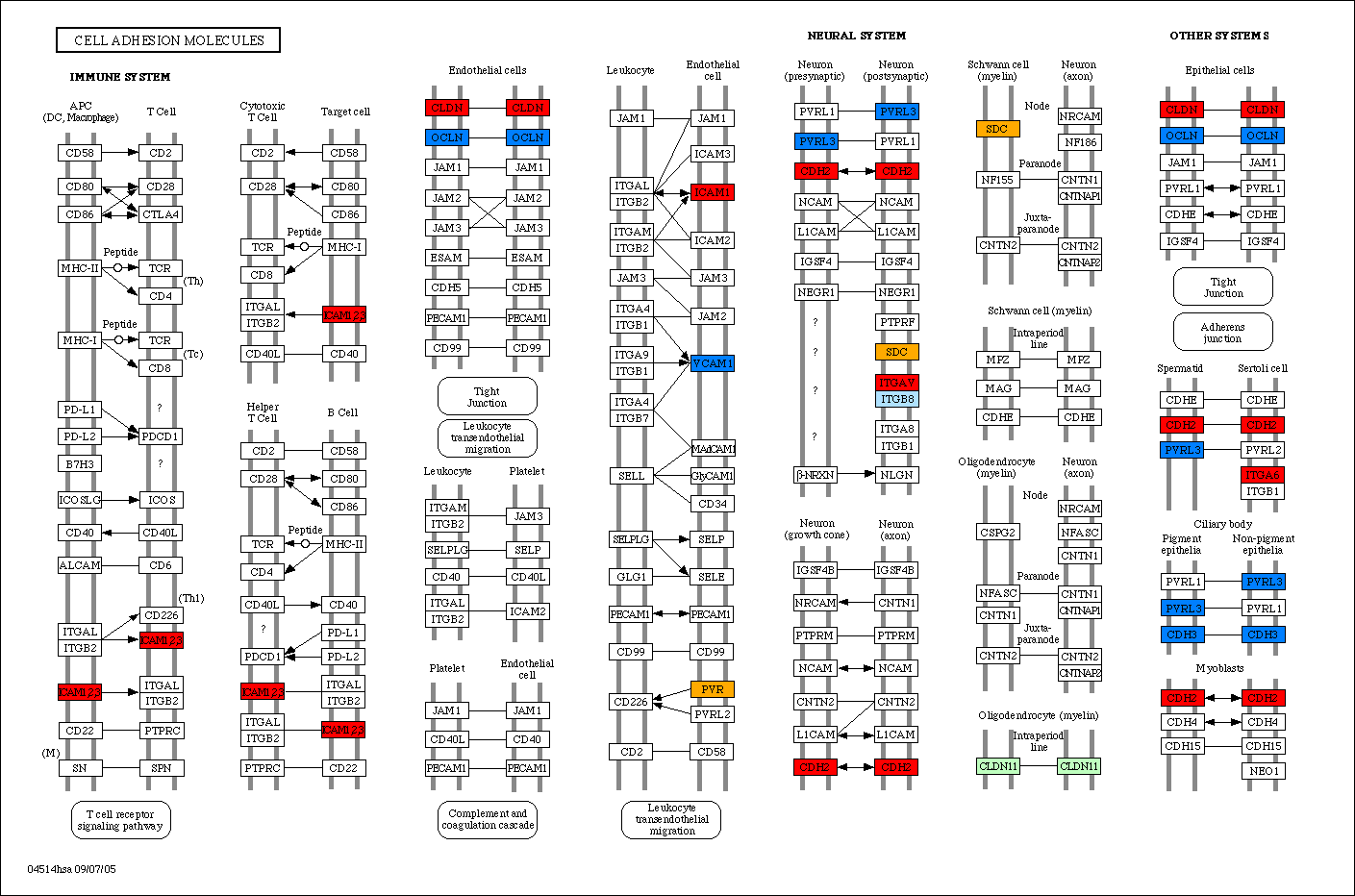


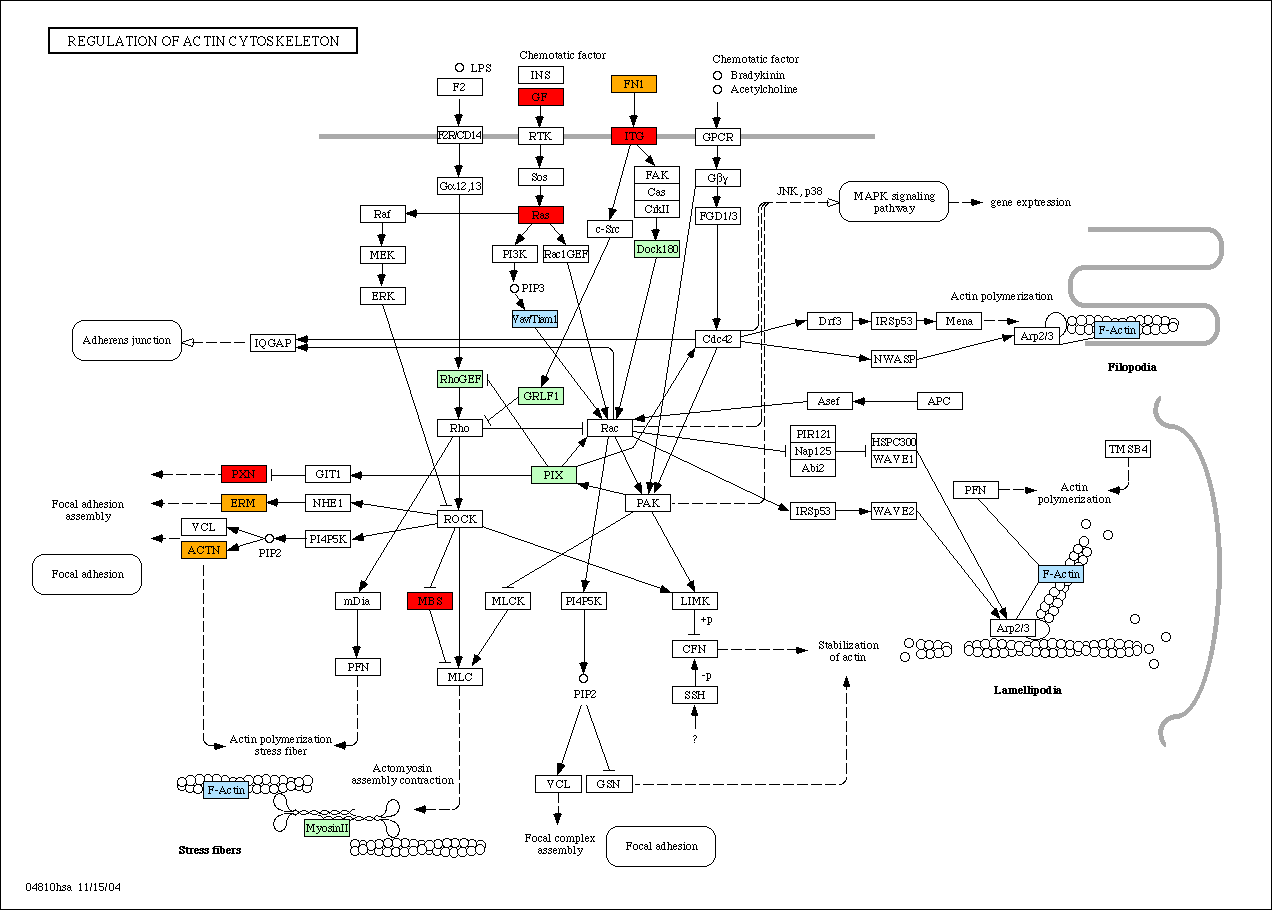


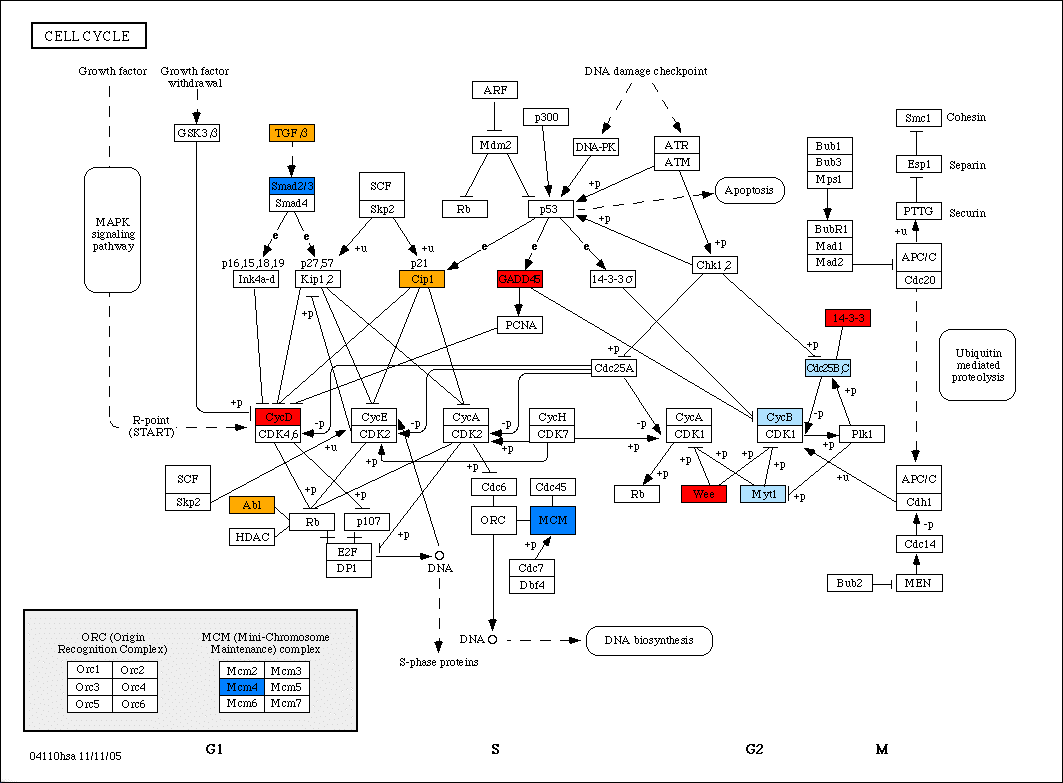


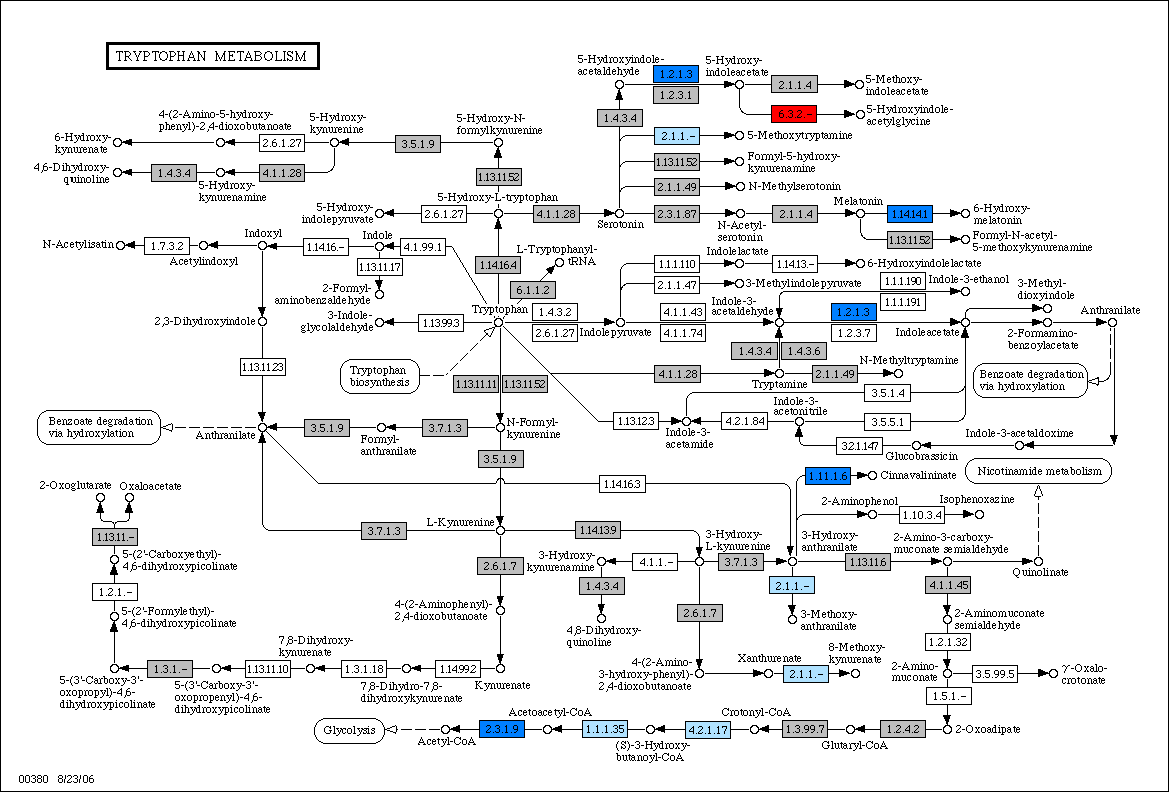


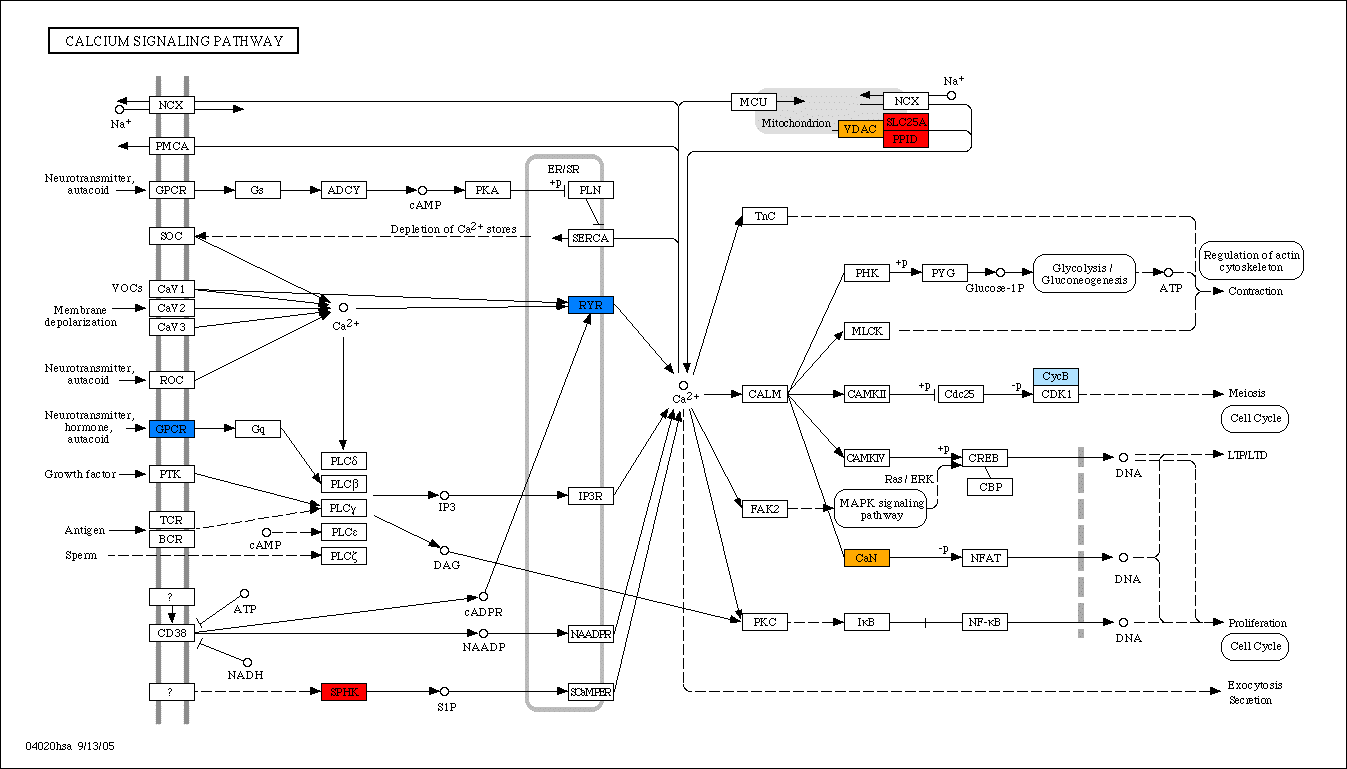


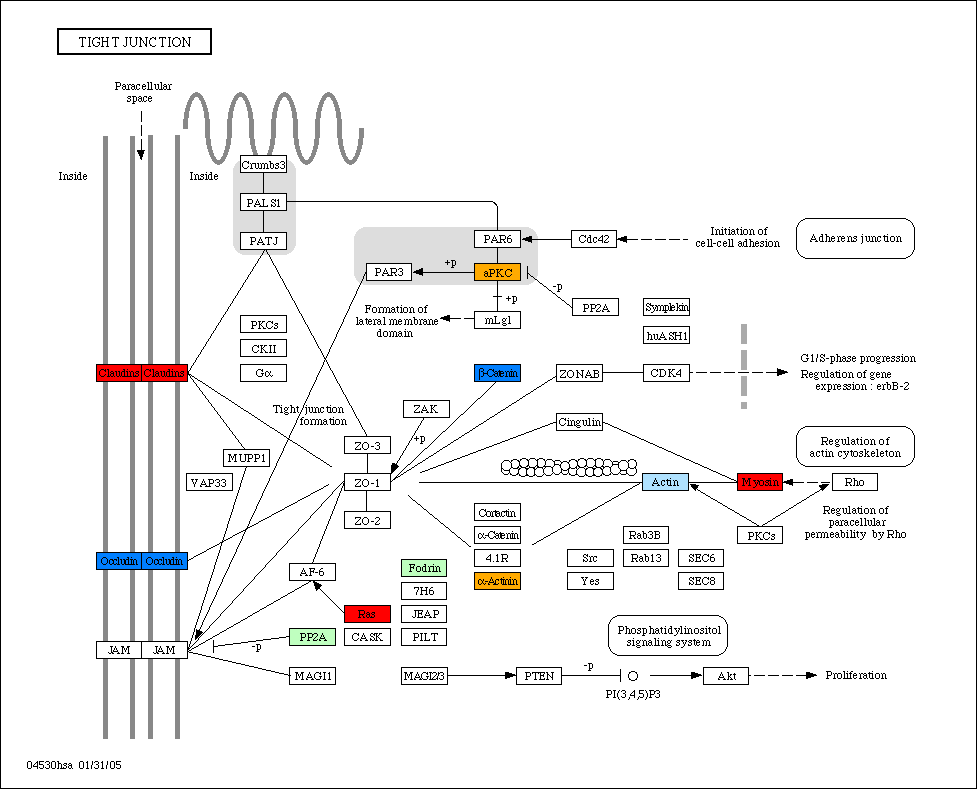


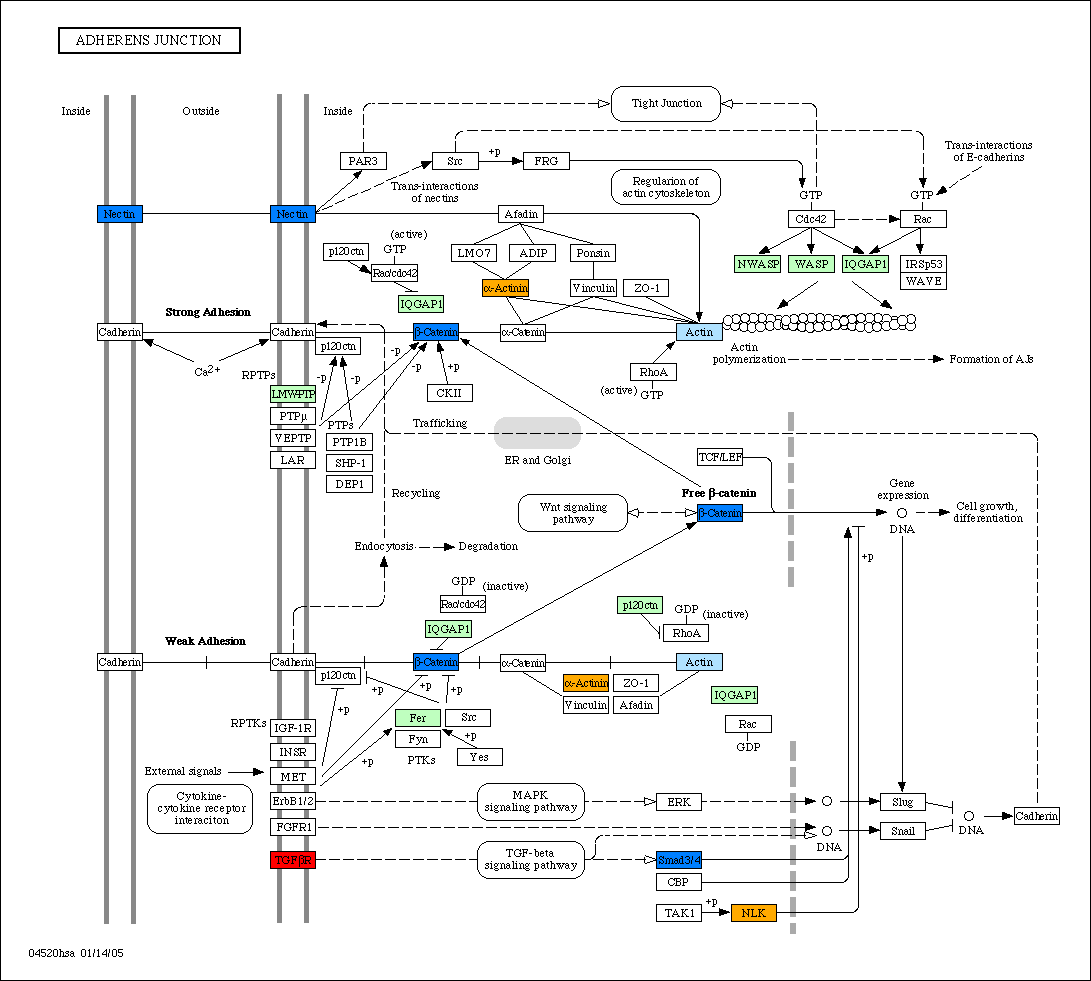


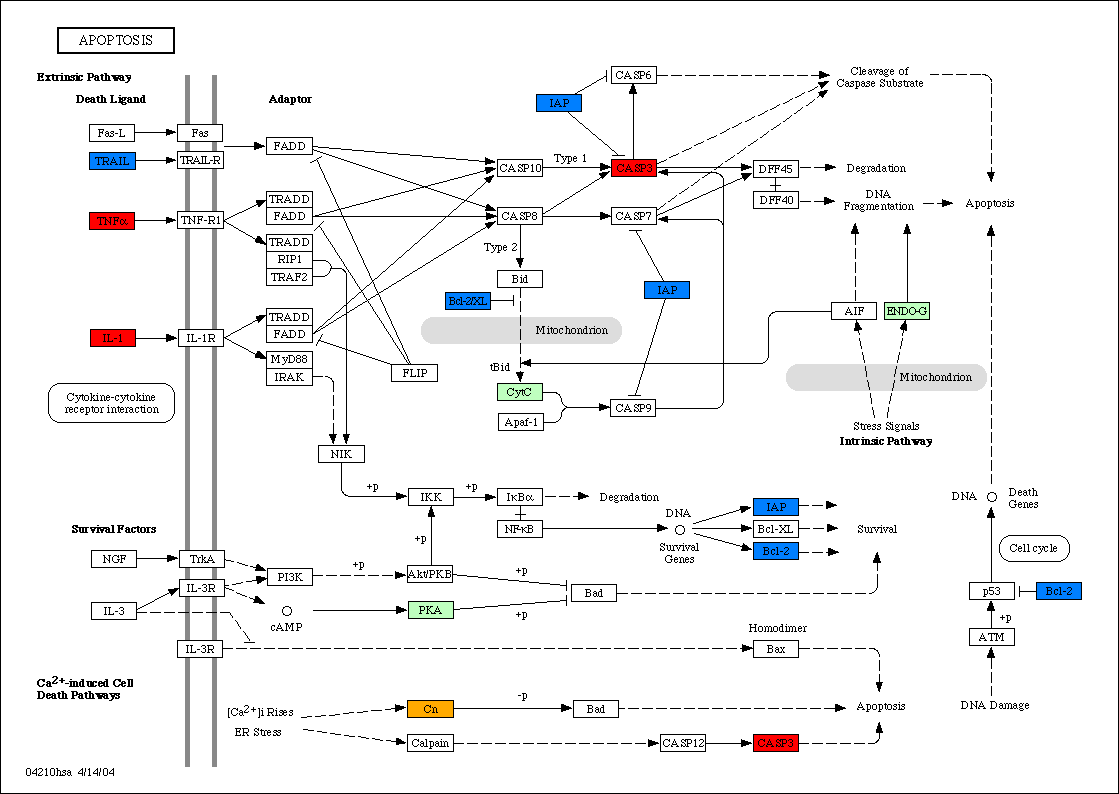

Supplement: Additional file 2 — KEGG analysis of differentially expressed genes. 267 genes in Additional file 1 with an EntrezGene ID were placed in the KEGG maps using the "color genes" option in the KEGG database [41]. Differentially expressed genes that only passed the SAM analysis were colored orange (up-regulated) and light-blue (down-regulated); genes that passed both statistical analyses (see Methods) were colored red (up-regulated) and blue (down-regulated). [file 1471-2164-8-383-S2.doc]
